# A mitofusin 2/HIF1α axis sets a maturation checkpoint in regenerating skeletal muscle

**DOI:** 10.1172/JCI161638

**Published:** 2022-12-01

**Authors:** Xun Wang, Yuemeng Jia, Jiawei Zhao, Nicholas P. Lesner, Cameron J. Menezes, Spencer D. Shelton, Siva Sai Krishna Venigalla, Jian Xu, Chunyu Cai, Prashant Mishra

**Affiliations:** 1 Children’s Medical Center Research Institute, University of Texas Southwestern Medical Center, Dallas, Texas, USA.; 2 Division of Hematology/Oncology, Boston Children’s Hospital, Boston, Massachusetts, USA.; 3 Department of Pediatric Oncology, Dana-Farber Cancer Institute, Harvard Medical School, Boston, Massachusetts, USA.; 4 Broad Institute of MIT and Harvard, Cambridge, Massachusetts, USA.; 5 Harold C. Simmons Comprehensive Cancer Center,; 6 Hamon Center for Regenerative Science and Medicine,; 7 Department of Pediatrics, and; 8 Department of Pathology, University of Texas Southwestern Medical Center, Dallas, Texas, USA.

**Keywords:** Muscle Biology, Stem cells, Epigenetics, Mitochondria

## Abstract

A fundamental issue in regenerative medicine is whether there exist endogenous regulatory mechanisms that limit the speed and efficiency of the repair process. We report the existence of a maturation checkpoint during muscle regeneration that pauses myofibers at a neonatal stage. This checkpoint is regulated by the mitochondrial protein mitofusin 2 (Mfn2), the expression of which is activated in response to muscle injury. Mfn2 is required for growth and maturation of regenerating myofibers; in the absence of Mfn2, new myofibers arrested at a neonatal stage, characterized by centrally nucleated myofibers and loss of H3K27me3 repressive marks at the neonatal myosin heavy chain gene. A similar arrest at the neonatal stage was observed in infantile cases of human centronuclear myopathy. Mechanistically, Mfn2 upregulation suppressed expression of hypoxia-induced factor 1α (HIF1α), which is induced in the setting of muscle damage. Sustained HIF1α signaling blocked maturation of new myofibers at the neonatal-to-adult fate transition, revealing the existence of a checkpoint that delays muscle regeneration. Correspondingly, inhibition of HIF1α allowed myofibers to bypass the checkpoint, thereby accelerating the repair process. We conclude that skeletal muscle contains a regenerative checkpoint that regulates the speed of myofiber maturation in response to Mfn2 and HIF1α activity.

## Graphical abstract

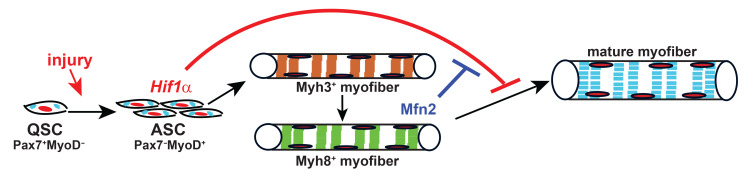

## Introduction

In response to injury, skeletal muscle undergoes a synchronized sequence of events over several days, including clearance of muscle debris, revascularization of the damaged region, activation and proliferation of adult muscle stem cells (MuSCs), fusion of MuSCs to form new myofibers, and maturation of new myofibers into adult fiber types (1). Promoting and accelerating muscle repair following injury has been a clinical goal for many years, and several supplements and therapies have been evaluated (2–4), although the underlying mechanisms are not well known. An understanding of how the individual steps in regeneration are coordinated would positively impact our ability to design therapies promoting efficient and rapid tissue regeneration in the setting of traumatic injury. To this end, a body of work has investigated requirements for efficient muscle repair, largely focusing on early events, including maintenance of the MuSC population through asymmetric division and niche factors, activation of MuSCs via expression of the myogenic regulatory factors and miRNAs, and modulation/clearance of the immune infiltrate (5–8).

In contrast, the requirements for the growth and maturation of myofibers during the late stages of muscle repair are relatively understudied, and whether the maturation stage of regeneration can be accelerated is unknown. During this latter stage, myofibers grow in size and sequentially express distinct myosin heavy chain (*Myh*) genes, transitioning from embryonic (*Myh3*) to neonatal (*Myh8*) to adult (*Myh1*, -*2*, -*4*, and -*7*) myosin expression. These myosin genes are functionally distinct, and confer contractile properties to the myofiber (9). Concomitant with these fate specification events, changes in mitochondrial content and morphology are apparent, and constitute a hallmark of myofiber maturation into adult muscle fibers (10–12). Establishment of a large mitochondrial population is critical to the health of adult myofibers; however, it is not known whether mitochondrial changes represent a passive characteristic of adult fiber specification, or whether the organelle directly regulates the myofiber maturation process itself.

Here, we investigated the role of mitochondria in impacting regeneration following muscle injury, focusing on the mitochondrial outer membrane protein mitofusin 2 (Mfn2). We found that Mfn2 is upregulated during MuSC activation, and is specifically required for the growth and adult fate specification in the latter stage of myofiber maturation. This finding is in contrast to the lack of a requirement for Mfn2 in fully differentiated muscle fibers. Mechanistically, regenerating *Mfn2*-knockout myofibers exhibited excess and sustained hypoxia-induced factor 1α (HIF1α) activity, which results in epigenetic alterations at the *Myh* locus, an accumulation of centrally nucleated growth-arrested myofibers, and a pause at the neonatal-to-adult fiber type transition. Interestingly, we observed a similar neonatal pause in pediatric cases of severe centronuclear myopathy (CNM). Further analysis in animal models revealed that elevated HIF1α signaling is sufficient to arrest regenerating myofibers at the neonatal-to-adult transition, thereby delaying the growth and maturation of regenerating muscle. During severe injury, we found that myofibers engage this regenerative checkpoint at the neonatal-to-adult fiber type transition that synchronizes the maturation of muscle fibers with the reestablishment of perfusion. Pharmacologic or genetic inhibition of HIF1α resulted in this checkpoint being bypassed, thereby accelerating the regenerative process. Together, these findings reveal a role for mitochondria and HIF1α in regulating late stages of regeneration, and inform on a strategy to accelerate muscle repair in response to traumatic injury.

## Results

### Induction of Mfn2 in activated MuSCs is required for growth of new myofibers.

**Figure 1:**
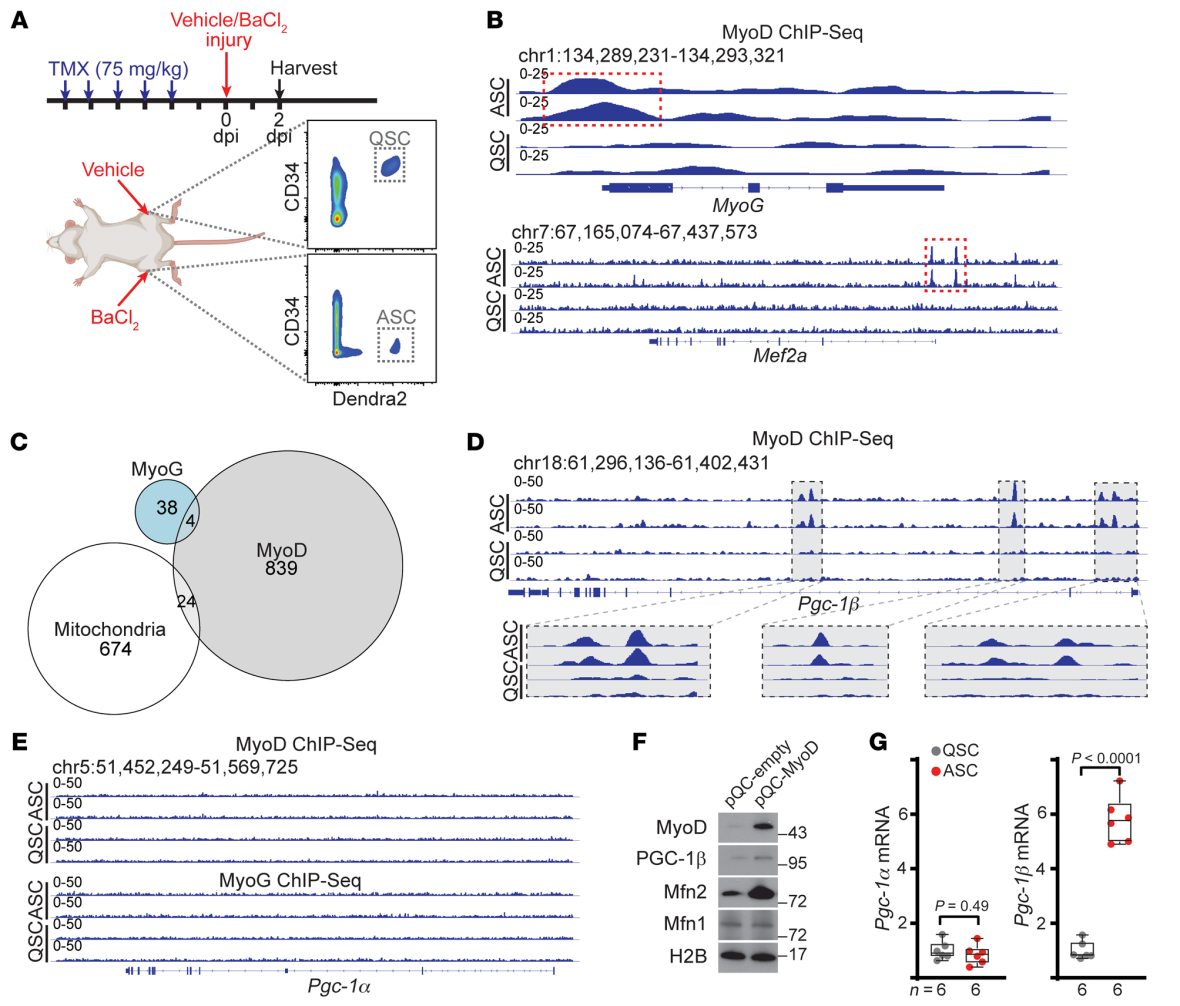
MyoD promotes expression of PGC-1β and mitochondrial genes in activated MuSCs. (**A**) Schematic of muscle injury experiments and FACS isolation of MuSCs. Tamoxifen was administered for 5 consecutive days to induce recombination, followed by BaCl_2_ (or vehicle) administration to induce muscle injury. Dendra2^+^DAPI^–^ MuSCs were collected at 2 days post injury (dpi). In vehicle-treated muscle, quiescent (CD34^+^) MuSCs (QSCs) were harvested. In injured muscle, activated (CD34^–^) MuSCs (ASCs) were harvested. (**B**) Representative snapshots of MyoD binding at the *MyoG* and *Mef2a* genes in 2-dpi QSCs and ASCs. Identified peaks in the proximity of the transcriptional start site are indicated by red boxes. (**C**) Venn diagram of MyoG- and MyoD-bound genes in 2-dpi ASCs. Genes were compared with a list of known mitochondrial regulators (Supplemental Table 1). (**D**) Representative snapshots of MyoD binding at the *Pgc-1β* gene in 2-dpi QSCs and ASCs. (**E**) Representative snapshots of MyoD and MyoG binding at the *Pgc-1α* gene in 2-dpi QSCs and ASCs. (**F**) Mouse 3T3-L1 fibroblasts were transfected with empty vector (pQC-empty) or MyoD-expressing vector (pQC-MyoD) and assessed by Western blotting 48 hours after transfection for the indicated targets. Histone 2B (H2B) is shown as a loading control. Molecular weight markers (in kDa) are indicated. (**G**) *Pgc-1α* and *Pgc-1β* mRNA levels (normalized to β2-microglobulin) assessed by qRT-PCR in wild-type QSCs and ASCs at 2 dpi. Statistical significance was assessed using 2-tailed *t* tests with adjustments for multiple comparisons (**G**). For each ChIP-seq data set, 3 biological replicates were analyzed. Box-and-whisker plots indicate median (horizontal line) and interquartile ranges (bounds of the box) from the indicated number of biological replicates; whiskers were plotted using Tukey’s method.

Recent results from in vitro–cultured C2C12 myoblasts and myotubes suggested that mitochondrial genes may be under the control of myogenic regulatory factors (13). We therefore performed ChIP sequencing (ChIP-seq) analysis to investigate whether mitochondrial genes are regulated by the master myogenic regulatory factors MyoG and MyoD during in vivo muscle regeneration. We injured tibialis anterior (TA) muscles of wild-type mice containing the MuSC-specific *Pax7-Cre^ERT2^* allele (14) driving conditional expression of a fluorescent and mitochondrially localized Dendra2 protein (15), which allows the facile detection and isolation of MuSCs and their progeny by FACS (Figure 1A). The TA muscle is routinely used in experimental studies of muscle regeneration, and exhibits robust activation of MuSCs, formation of new myofibers, and complete functional recovery in response to muscle injury (16, 17). In noninjured vehicle-injected muscle, quiescent Dendra2^+^ MuSCs (QSCs) were largely CD34^+^, a marker of quiescence in this lineage, while in 2 day post injury (dpi) muscle, Dendra2^+^ MuSCs lost CD34 expression, indicating an activated state (ASCs) (18) (Figure 1A). ChIP-seq experiments from these 2 populations allowed the identification of differentially bound peaks in QSCs versus ASCs; this analysis revealed binding of MyoG and MyoD to candidate regulatory elements for 42 and 867 genes (respectively) specific to ASCs (FDR < 0.05; Supplemental Table 1; supplemental material available online with this article; https://doi.org/10.1172/JCI161638DS1), including previously validated targets (Figure 1B). We compared our identified MyoD and MyoG targets in ASCs against a list of known regulators of mitochondrial biology (Supplemental Table 1; 696 genes). MyoG was not bound to any mitochondrial genes, and MyoD was bound to 24 mitochondrial genes (Supplemental Table 1 and Figure 1C), including the master mitochondrial regulator *Pgc-1β*. MyoD bound in vivo to 3 discrete genomic regions within the *Pgc-1β* gene, including peaks in the proximity of the promoter and intron 1 (Figure 1D). We did not detect binding of MyoD or MyoG to the related family member, *Pgc-1α* (Figure 1E), and overexpression of MyoD in mouse 3T3 fibroblasts was sufficient to induce expression of *Pgc-1β* (Figure 1F). Correspondingly, we found that *Pgc-1β* transcripts were significantly induced in vivo in ASCs versus QSCs, while *Pgc-1α* transcripts were unchanged (Figure 1G).

**Figure 2:**
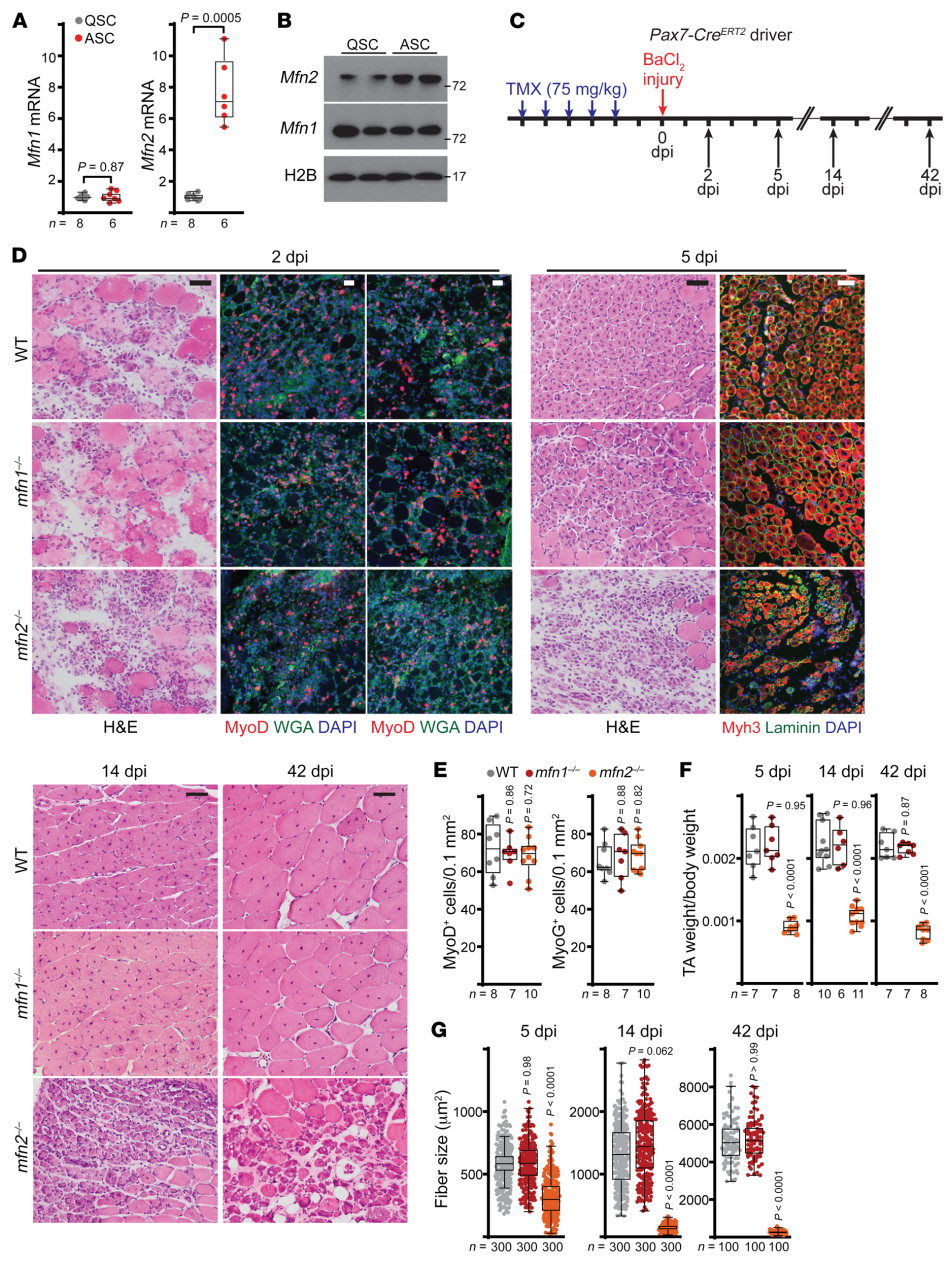
Mfn2 is required for growth of regenerating myofibers. (**A**) *Mfn1* and *Mfn2* transcript levels (normalized to β2-microglobulin) in wild-type QSCs and 2-dpi ASCs. (**B**) Western blots for Mfn1 and Mfn2 in wild-type QSCs and 2-dpi ASCs. Histone 2B (H2B) is displayed as a loading control; molecular weight (kDa) is indicated. (**C**) Schematic of muscle injury experiments. Tamoxifen (TMX) was given for 5 consecutive days to induce recombination, followed by BaCl_2_-mediated muscle injury. Muscles were analyzed at the indicated time points. (**D**) Representative histology (H&E) and immunofluorescence images of muscle cross sections of the indicated genotypes and time points. Staining for nuclei (DAPI, blue), myofiber boundaries (wheat germ agglutinin [WGA, green] or laminin [green]), and MyoD, MyoG, or Myh3 (red) is presented. Scale bars: 50 μm. (**E**) MyoD^+^ and MyoG^+^ cell numbers from 2-dpi muscles, normalized to cross-sectional area. (**F**) Tibialis anterior (TA) muscle weight (normalized to body weight) from mice at the indicated time points. (**G**) Cross-sectional area of regenerating fibers from muscles at 5, 14, and 42 dpi. *n* = 100–300 myofibers analyzed from 6–11 mice per group. Statistical significance was assessed using 2-tailed *t* test (**A**), 1-way ANOVA (**E** and **F**), or Kruskal-Wallis test (**G**) with adjustments for multiple comparisons. *P* values reflect comparison with the wild-type group. Box-and-whisker plots indicate median (horizontal line) and interquartile ranges (bounds of the boxes) from the indicated number of biological replicates; whiskers were plotted using Tukey’s method.

PGC-1β has been established to promote transcription of genes involved in numerous mitochondrial processes, including biogenesis, oxidative phosphorylation, and mitochondrial dynamics (19). In particular, PGC-1β promotes transcription of the *Mfn* genes, which are localized to the mitochondrial outer membrane and responsible for initiation of mitochondrial fusion, as well as calcium homeostasis (20–22). Indeed, exogenous PGC-1β overexpression in vitro is selectively associated with Mfn2 (but not Mfn1) accumulation (Figure 1F). We found that *Mfn2*, but not *Mfn1*, was selectively induced in ASCs versus QSCs (Figure 2, A and B), suggesting a specific role for Mfn2 in muscle regeneration. We therefore made use of conditional knockout alleles for *Mfn1* or *Mfn2* (23), combined with the *Pax7-Cre^ERT2^* driver, to deplete Mfn1 or Mfn2 levels in MuSCs (Supplemental Figure 1, A and B) and assess their role in muscle regeneration. Following tamoxifen-induced depletion and muscle injury (Figure 2C), animals with *Mfn2*-deleted MuSCs (hereafter, *mfn2^–/–^*) were able to generate MyoD^+^ and MyoG^+^ ASCs at 2 dpi (Figure 2, D and E). At 5 dpi, *mfn2^–/–^* animals formed a large number of de novo myofibers, identified by expression of the embryonic myosin heavy chain (*Myh3*) and prominent centralized nuclei; however, the mutant myofibers were significantly diminished in size, and the injured muscle area retained a significant amount of interstitial tissue (Figure 2, D and F). At 14 and 42 dpi, new myofibers (marked by centralized nuclei) from wild-type animals continued to grow in size; however, myofibers and muscle in *mfn2^–/–^* animals remained significantly smaller with retained centralized nuclei (Figure 2, D, F, and G). In contrast, animals with *Mfn1*-deleted MuSCs (hereafter *mfn1^–/–^*) displayed no defects in stem cell activation, myofiber formation, fiber growth, or muscle growth (Figure 2, D–G).

The above results indicate that Mfn2 is not required in vivo for MuSC activation and fusion to form new myofibers, but is necessary for myofiber growth after fusion. We investigated the requirement for Mfn2 in vitro, making use of primary MuSCs purified from tamoxifen-treated animals. *Mfn2^–/–^* MuSCs exhibited no defects in their ability to generate a membrane potential in response to mitochondrial substrates (Supplemental Figure 2, A and B). Additionally, we did not observe elevated mitochondrial superoxide levels (Supplemental Figure 2, C and D) or defects in oxygen consumption rates (Supplemental Figure 2E). Proliferation of MuSCs was unaffected by Mfn2 loss (Supplemental Figure 2F), and *mfn2^–/–^* MuSCs were able to fuse and form myotubes at similar rates to wild-type MuSCs (Supplemental Figure 2, G and H). Thus, Mfn2 is dispensable for myoblast proliferation and differentiation in vitro, similar to our observations in vivo.

**Figure 3:**
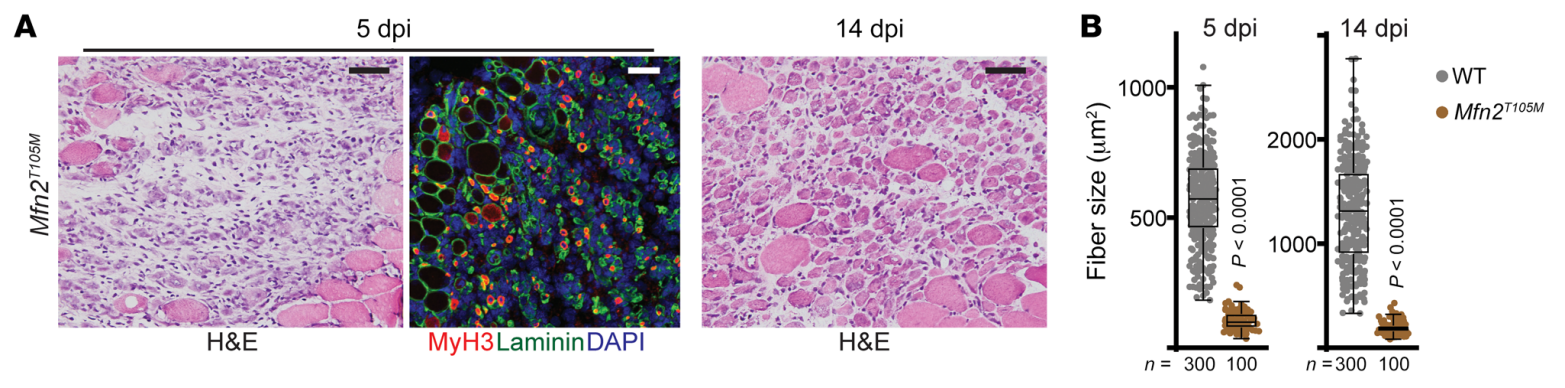
The CMT2A *Mfn2^T105M^* allele inhibits growth of regenerating myofibers. (**A**) Representative histology (H&E) and immunofluorescence images of muscle cross sections from *Mfn2^T105M^* mice at 5 and 14 dpi. Myh3 (red), laminin (green), and DAPI (blue) staining are presented. Scale bars: 50 μm. (**B**) Cross-sectional area of regenerating fibers from muscles at 5 and 14 dpi. *n =* 100–300 myofibers analyzed from 6–10 mice per group. Statistical significance was assessed using the Kolmogorov-Smirnov test (**B**). *P* values reflect comparison with the wild-type group. Box-and-whisker plots indicate median (horizontal line) and interquartile ranges (bounds of the boxes) from the indicated number of biological replicates; whiskers were plotted using Tukey’s method.

*MFN2* mutations in humans are a common cause of Charcot-Marie-Tooth disease (type 2A), an autosomal dominant axonal neuropathy with associated muscle atrophy (24, 25). We tested the effect of the disease-associated T105M mutation, making use of a Cre-inducible *Mfn2^T105M^* allele inserted into the Rosa26 locus (26). Like *mfn2^–/–^* animals, expression of the dominant negative *Mfn2^T105M^* did not inhibit MuSC activation or myofiber formation, but severely restricted the growth of new myofibers (Figure 3, A and B, and Supplemental Figure 1, C–E).

### Regenerating mfn2^–/–^ myofibers arrest at the neonatal stage.

**Figure 4:**
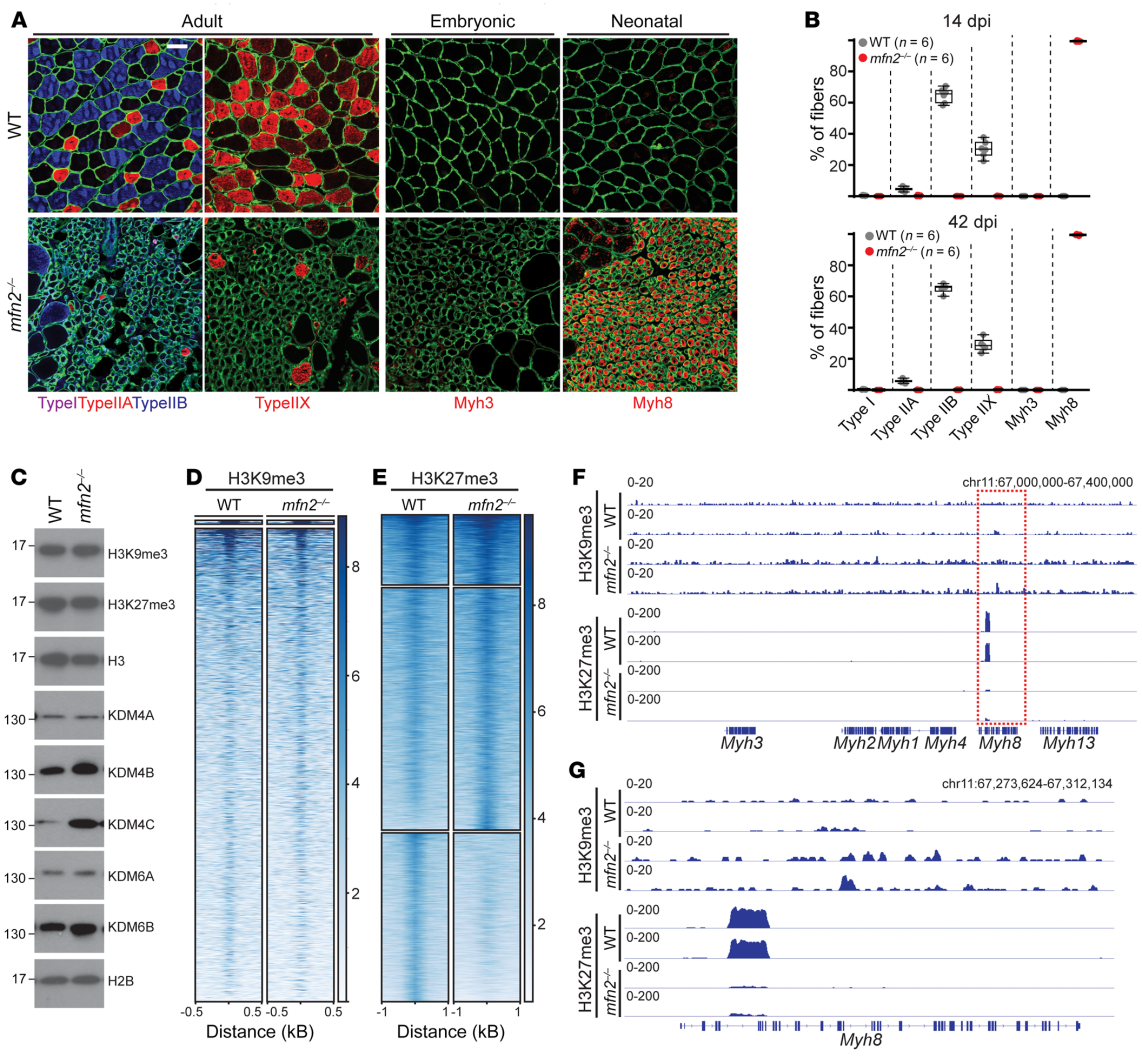
Regenerating *mfn2^–/–^* myofibers are arrested at a neonatal fate. (**A**) Representative immunofluorescence images of muscle cross sections from mice of the indicated genotype at 14 dpi. Sections were stained with antibodies targeting fiber-type-specific myosin heavy chains, including Myh7 (type I, purple), Myh2 (type IIa, red), Myh4 (type IIb, blue), Myh1 (type IIx, red), Myh3 (embryonic, red), and Myh8 (neonatal, red). Myofiber borders were visualized with laminin staining (green). Scale bar: 50 μm. (**B**) Quantification of fiber types (as a percentage of total regenerating fibers) in wild-type and *mfn2^–/–^* animals at 14 and 42 dpi. (**C**) Levels of H3K9me3, H3K27me3, and a number of KDM family members in 14-dpi myofibers. Molecular weight markers (in kDa) are indicated. Histone 2B (H2B) and histone 3 (H3) are shown as loading controls. (**D**) Heatmaps representing normalized H3K9me3 ChIP-seq intensities of identified genome-wide peaks in 14-dpi myofibers of the indicated genotype, after k-means clustering. Peaks were ranked according to their ChIP-seq intensity in wild-type samples. *n =* 3 mice per group. (**E**) Heatmaps representing normalized H3K27me3 ChIP-seq intensities of identified genome-wide peaks in 14 dpi myofibers of the indicated genotype, after k-means clustering. Peaks were ranked according to their ChIP-seq intensity in wild-type samples. *n =* 3 mice per group. (**F**) Representative snapshots for H3K9me3 and H3K27me3 ChIP-seq analyses performed in 14-dpi myofibers of the indicated genotype, focusing on the myosin heavy chain locus. Increased deposition of H3K27me3 at the *Myh8* gene is highlighted (red box). (**G**) Representative snapshots of H3K9me3 and H3K27me3 deposition at the *Myh8* gene in 14-dpi myofibers of the indicated genotype. For each ChIP-seq data set, 3 biological replicates were analyzed. Box-and-whisker plots indicate median (horizontal line) and interquartile ranges (bounds of the boxes) from the indicated number of biological replicates; whiskers were plotted using Tukey’s method.

During regenerative growth, new myofibers proceed from an embryonic to neonatal to adult fate, classified by sequential expression of *Myh* genes localized at the *Myh* locus (27–29). By 14 dpi, wild-type myofibers have taken on adult fates indicated by a mixture of adult type I (Myh7), type IIa (Myh2), type IIx (Myh1), and type IIb (Myh4) fiber types, and a lack of embryonic (Myh3) and neonatal (Myh8) fiber types (Figure 4, A and B). The growth-arrested *mfn2^–/–^* myofibers do not adopt an adult fate, as evidenced by a lack of adult Myh expression; instead the vast majority of new fibers adopt a neonatal Myh8^+^ fate (Figure 4, A and B). The mechanisms underlying sequential expression of *Myh* genes are not currently understood, but potentially rely on epigenetic histone marks, which have been previously implicated in MuSC maintenance (30–32). Western blot analysis of 14-dpi regenerating myofibers revealed upregulation of a subset of lysine demethylase (KDM) family members, including KDM4 and KDM6 family members (Figure 4C). As these enzymes exhibit H3K9 and H3K27 demethylase activity, respectively, we therefore examined H3K9me3 and H3K27me3 deposition in 14-dpi wild-type and *mfn2^–/–^* myofibers. Global levels of H3K9me3 and H3K27me3 were not significantly affected (Figure 4C). To more precisely examine genome-wide deposition, we performed ChIP-seq analysis in 14-dpi wild-type and *mfn2^–/–^* regenerating myofibers. H3K9me3 deposition was observed at expected loci based on previous reports (33, 34) (Supplemental Figure 3A), and k-means clustering revealed similar deposition patterns between wild-type and *mfn2^–/–^* regenerating myofibers (Figure 4D and Supplemental Table 2). H3K27me3 is deposited by polycomb gene (PcG) complexes, and evaluation of H3K27me3 deposition revealed similar patterns at known PcG-binding genes (35) between wild-type and *mfn2^–/–^* myofibers (Supplemental Figure 3, B and C). However, k-means analysis of genome-wide H3K27me3 peaks revealed a cluster with significantly decreased deposition in *mfn2^–/–^* myofibers (Figure 4E), which included peaks in the *Myh8* locus (Supplemental Table 2). Examination of the *Myh* locus revealed significant deposition of H3K27me3 marks in an intragenic region of the *Myh8* locus in wild-type 14-dpi myofibers (Figure 4, F and G). Intragenic deposition of H3K27me3 is highly associated with repressed transcription (36, 37). Interestingly, the H3K27me3 peak at the *Myh8* locus was significantly reduced in *mfn2^–/–^* regenerating myofibers (Figure 4, F and G). This identified region spanned multiple introns, and was verified by ChIP-qPCR in independent experiments (Supplemental Figure 3, D and E). No significant deposition of H3K27me3 was noted at other *Myh* genes, and no deposition of H3K9me3 was observed within the *Myh* locus (Figure 4F). We also did not observe differential deposition or enrichment of H3K9me3 or H3K27me3 in MyoD or MyoG targeted regions (Supplemental Figure 3F). Thus, the observed maturation arrest at the neonatal (Myh8^+^) stage observed in *mfn2^–/–^* regenerating myofibers correlates with loss of repressive H3K27me3 marks at the *Myh8* locus.

### Sustained HIF1α signaling underlies the regenerative arrest in mfn2^–/–^ myofibers.

**Figure 5:**
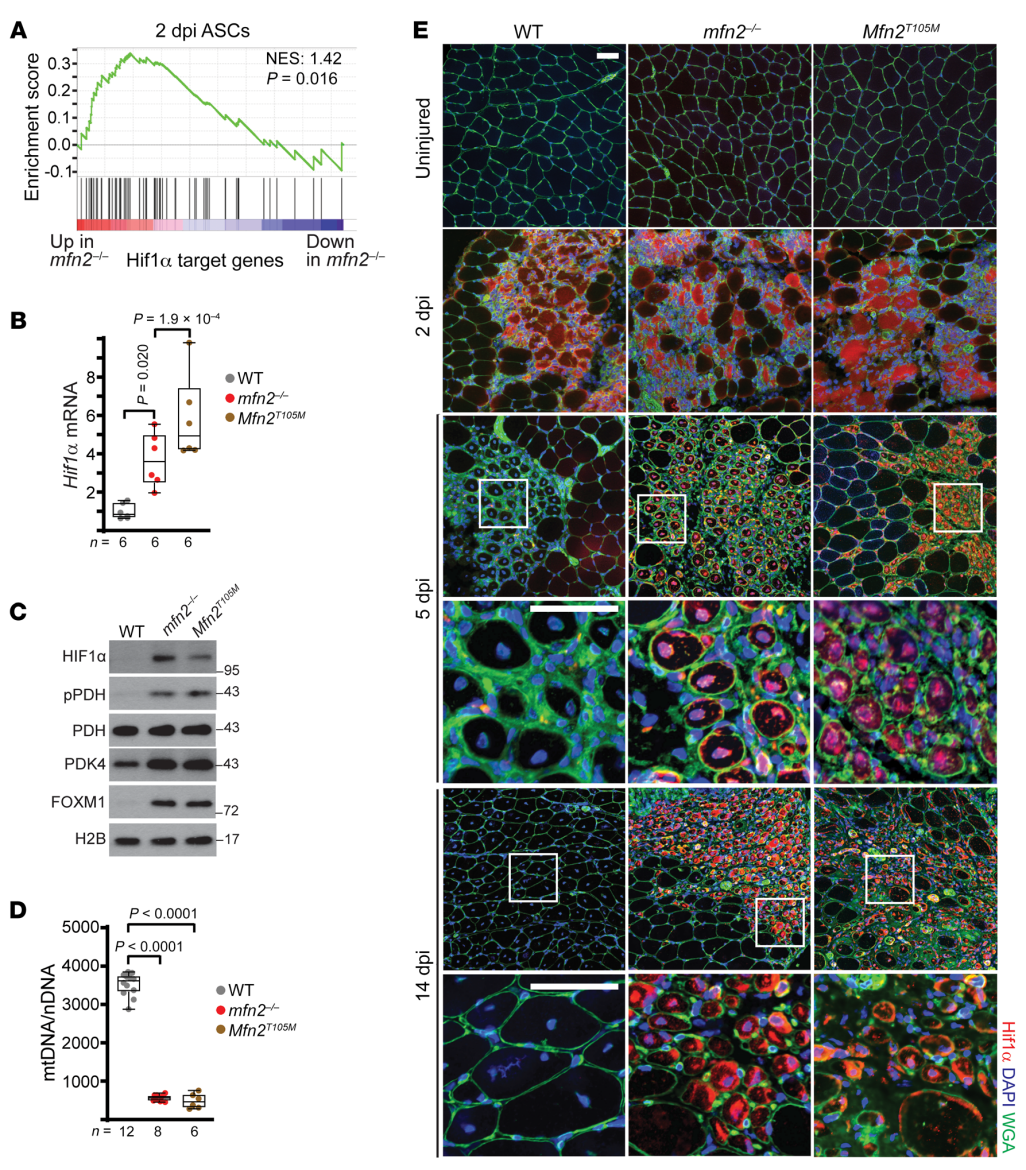
*Mfn2*-mutant regenerating myofibers exhibit sustained HIF1α signaling. (**A**) Gene set enrichment analysis of HIF1α target genes in *mfn2^–/–^* versus wild-type ASCs. NES, normalized enrichment score. (**B**) *Hif1α* mRNA (normalized to β2-microglobulin) in 14-dpi myofibers. (**C**) Western blot analysis of the indicated proteins in 14-dpi myofibers. Molecular weights (kDa) are indicated. (**D**) Mitochondrial genome (mtDNA) content, normalized to nuclear genome content (nDNA) in 14-dpi myofibers. (**E**) Representative immunofluorescence images of HIF1α (red), nuclei (DAPI, blue), and myofiber boundaries (wheat germ agglutinin [WGA], green) in muscle cross sections at indicated time points. Scale bars: 50 μm. Statistical significance was assessed using 1-way ANOVA (**B** and **D**) with adjustments for multiple comparisons. Box-and-whisker plots indicate median (horizontal line) and interquartile ranges (bounds of the boxes) from the indicated number of biological replicates; whiskers were plotted using Tukey’s method.

To examine the underlying mechanisms regarding regulation of the neonatal-to-adult transition in *mfn2^–/–^* animals, we employed RNA-seq to compare wild-type and *mfn2^–/–^* ASCs (Supplemental Figure 4A and Supplemental Table 3). Gene ontology analysis of downregulated genes revealed enrichment in developmental pathways, consistent with the observed effects on myofiber development (Supplemental Figure 4B and Supplemental Table 3). In contrast, genes upregulated in *mfn2^–/–^* ASCs were enriched for metabolic pathways, which included the transcriptional upregulation of HIF1α (Supplemental Figure 4, A and C, and Supplemental Table 3). Indeed, gene set enrichment analysis indicated that HIF1α targets were significantly enriched among upregulated genes in *mfn2^–/–^* ASCs (Figure 5A). At 14 dpi, *mfn2^–/–^* and *Mfn2^T105M^* regenerating myofibers exhibited continued upregulation of *Hif1α* transcripts and protein, as well as increased levels of HIF1α targets (including the histone demethylases KDM4B, KDM4C, and KDM6B), increased phosphorylation of pyruvate dehydrogenase (PDH), and impaired mitochondrial biogenesis (Figure 4C and Figure 5, B–D); these effects are consistent with known roles of HIF1α in regulation of mitochondrial biology (38). To further investigate consequences of these arrested myofibers, we performed steady-state metabolomics measurements in wild-type and *mfn2^–/–^* regenerating myofibers at 5 and 14 dpi. Unsupervised hierarchical clustering separated mature 14-dpi wild-type myofibers; however, the metabolic profiles of 14-dpi *mfn2^–/–^* myofibers were interspersed with the 5-dpi immature myofibers (Supplemental Figure 5A). We identified a number of metabolites altered in *mfn2^–/–^* myofibers at 5 and 14 dpi (Supplemental Figure 5B and Supplemental Table 4). Overall, we did not observe changes in most TCA cycle metabolites (Supplemental Figure 5C). Interestingly, metabolites related to ketone and amino acid oxidation (acetoacetate, β-hydroxybutyrate, glutarylcarnitine) were significantly upregulated in *mfn2^–/–^* myofibers at both 5 and 14 dpi (Supplemental Figure 5C).

We examined HIF1α protein levels in vivo during muscle regeneration in wild-type, *mfn2^–/–^*, and *Mfn2^T105M^* animals. Consistent with previous reports (39), wild-type animals exhibited a significant increase in HIF1α protein levels 2 days after injury within the damaged region of muscle (Figure 5E). By 5 dpi, HIF1α levels were largely reduced to preinjury levels, indicating that the injury-induced rise in HIF1α is transient in wild-type animals (Figure 5E). Injured *mfn2^–/–^* and *Mfn2^T105M^* animals also display a significant upregulation of HIF1α at 2 dpi, and HIF1α levels remained high and present in the nuclei of new myofibers at 5 and 14 dpi (Figure 5E). This was not due to impaired vascularization of the regenerating region, based on CD31 staining for capillaries at 14 dpi (Supplemental Figure 4D). Thus, Mfn2 is required after injury to lower HIF1α levels in later stages of regeneration. Mfn2 has been previously shown to regulate localization of NFATC2 (NFAT1), a calcium-dependent transcription factor (40). At 5 dpi and 14 dpi, *mfn2^–/–^* and *Mfn2^T105M^* regenerating myofibers displayed substantial increases in nuclear localization of NFATC2 (Supplemental Figure 4E). Transcription of *Hif1α* has been previously suggested to be induced by altered calcium levels (41), and we found that NFATC2 overexpression was sufficient to induce HIF1α protein levels in hypoxia-treated cells in vitro (Supplemental Figure 4F). We also observed NFATC2 nuclear localization and stimulated transcription of *Hif1α* in *mfn2^–/–^* myotubes in vitro (Supplemental Figure 2, I and J). We therefore performed in vivo ChIP-seq analysis of NFATC2 binding regions, which indicated significantly increased occupancy of NFATC2 at the *Hif1α* promoter in 14-dpi *mfn2^–/–^* myofibers (Supplemental Figure 4G). We did not observe changes in methylation marks at either *Hif1α* or *NFATC2* loci (Supplemental Figure 6, A and B). These data support a model whereby upregulation of Mfn2 during muscle regeneration negatively regulates NFATC2 activity in order to suppress *Hif1α* transcription induced by muscle injury; however, we cannot rule out the possibility of alternative mechanisms for HIF1α stabilization.

**Figure 6:**
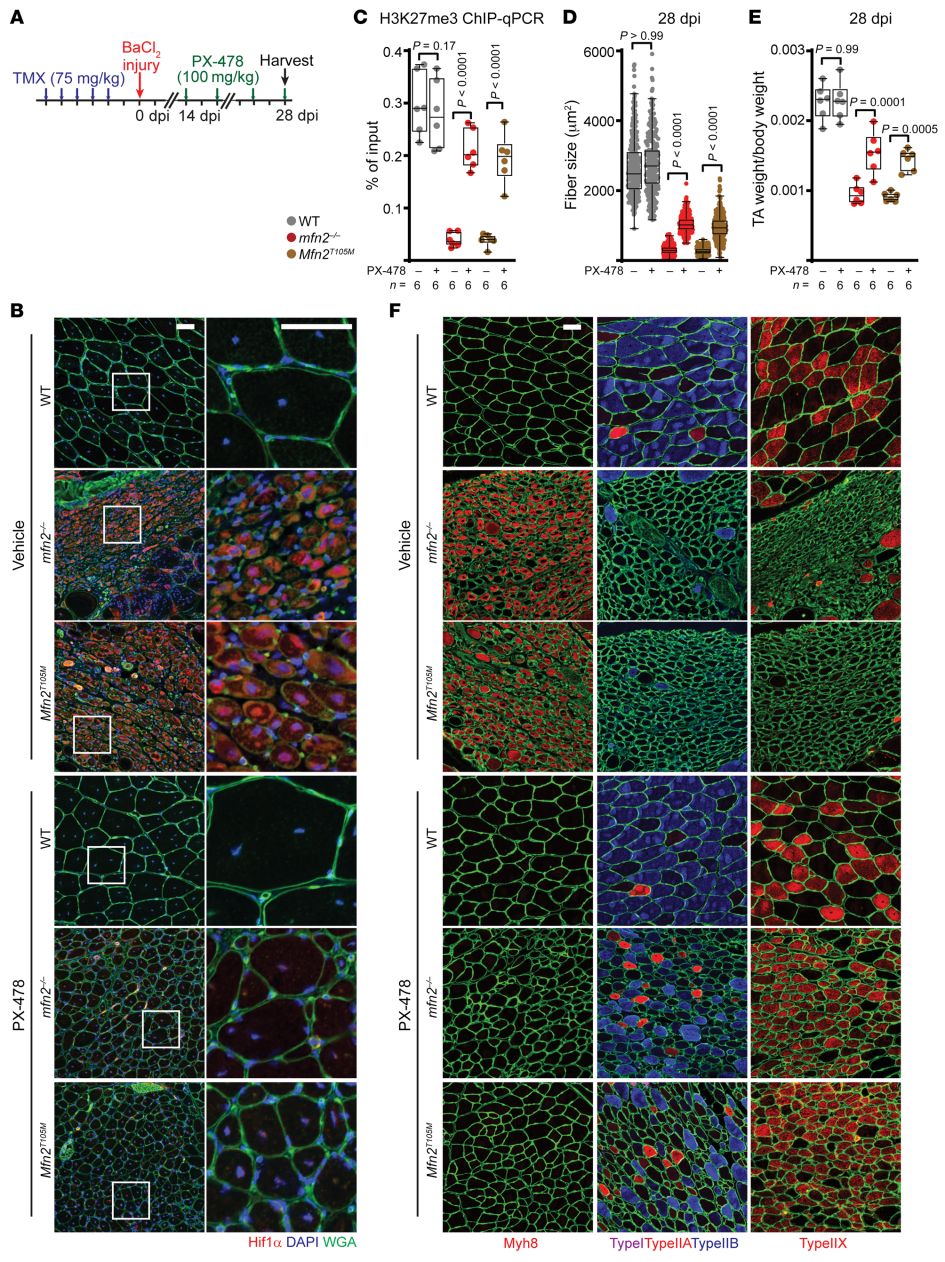
HIF1α inhibition enables maturation of *Mfn2*-mutant regenerating myofibers. (**A**) Schematic of PX-478 experiment. Tamoxifen (TMX) administration (5 consecutive days) was followed by BaCl_2_-induced muscle injury. At 14–28 dpi, mice were treated with PX-478 (or vehicle). (**B**) Representative immunofluorescence images of HIF1α (red), nuclei (DAPI, blue), and myofiber boundaries (wheat germ agglutinin [WGA], green) in 28-dpi muscle cross sections of the indicated genotype and treatment. Scale bars: 50 μm. (**C**) Enrichment (% of input) from H3K27me3 ChIP-qPCR experiments targeting *Myh8* in 28-dpi myofibers. (**D**) Cross-sectional area of 28-dpi myofibers of the indicated genotype and treatment. *n =* 300 myofibers analyzed from 6 mice per group. (**E**) TA muscle weight (normalized to body weight) of the indicated genotype and treatment at 28 dpi. (**F**) Representative immunofluorescence images of 28-dpi muscle cross sections of the indicated genotype and treatment. Sections were stained with antibodies targeting fiber-type-specific myosin heavy chains: Myh7 (type I, purple), Myh2 (type IIa, red), Myh4 (type IIb, blue), Myh1 (type IIx, red), Myh8 (neonatal, red), and myofiber boundaries (laminin, green). Scale bar: 50 μm. Statistical significance was assessed using 2-way ANOVA (**C** and **E**) or Kruskal-Wallis (**D**) test with adjustments for multiple comparisons. Box-and-whisker plots indicate median (horizontal line) and interquartile ranges (bounds of the boxes) from the indicated number of biological replicates; whiskers were plotted using Tukey’s method.

HIF1α signaling has been previously linked to alterations in histone methylation, via the induction of demethylases or regulation of the PRC2 complex (42–44). We therefore tested whether excess HIF1α signaling mediates the neonatal (Myh8^+^) arrest observed in regenerating *Mfn2*-mutant myofibers. We first arrested *mfn2^–/–^* or *Mfn2^T105M^* myofibers in the Myh8^+^ state at 14 dpi, and then treated animals for an additional 2 weeks with vehicle or PX-478, a compound known to reduce HIF1α levels (45, 46) (Figure 6A). PX-478 treatment for 14 days was sufficient to lower HIF1α levels in both *mfn2^–/–^* and *Mfn2^T105M^* myofibers (Figure 6B). ChIP-qPCR analysis targeting the *Myh8* allele revealed that PX-478 treatment restored H3K27me3 deposition at the *Myh8* locus (Figure 6C). Strikingly, we observed that PX-478 (but not vehicle) treatment was sufficient to release *mfn2^–/–^* and *Mfn2^T105M^* myofibers from their neonatal arrested state, allowing them to now adopt adult fates (Figure 6F). This was accompanied by a partial rescue of fiber and muscle size (Figure 6, D and E) by 28 dpi. Thus, inhibition of HIF1α signaling is sufficient to release myofibers from the maturation arrest in these *Mfn2*-mutant animal models. To test the role of H3K27me3 demethylases, we repeated these experiments, but instead treated animals with GSK-J4, a potent inhibitor of KDM6A and KDM6B demethylases (47). Two-week treatment starting at 14 dpi (Supplemental Figure 7A) was sufficient to restore H3K27me3 deposition at the *Myh8* locus (Supplemental Figure 7B). We observed that GSK-J4–treated animals were able to proceed through the neonatal stage and express adult myosins (Supplemental Figure 7C), accompanied by increased fiber size and muscle growth (Supplemental Figure 7, D and E). Thus, inhibition of H3K27 demethylases regulates procession through the neonatal stage during muscle regeneration in our *Mfn2*-mutant animal models.

### Excess HIF1α is sufficient to arrest regenerating myofibers at the neonatal-adult transition.

**Figure 7:**
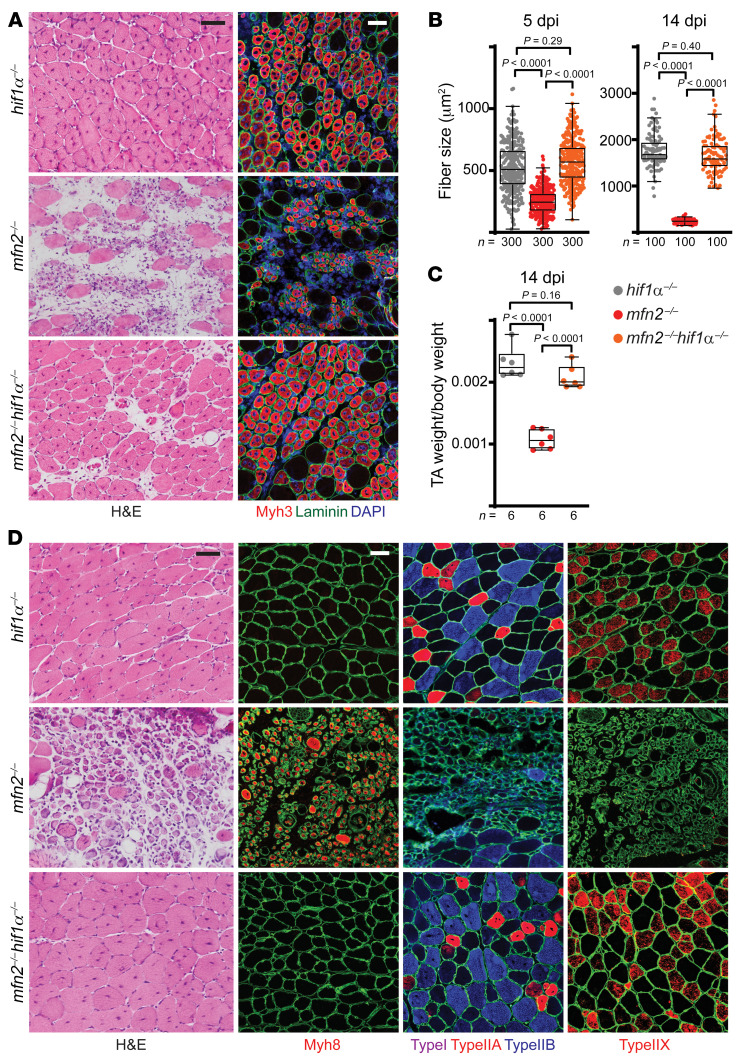
*Hif1α* deletion enables maturation in *mfn2^–/–^* regenerating myofibers. (**A**) Representative histology (H&E) and immunofluorescence images of muscle cross sections (5 dpi). Muscle cross sections were stained for Myh3 (red), nuclei (DAPI, blue), and myofiber boundaries (laminin, green). Scale bars: 50 μm. (**B**) Cross-sectional area of regenerating myofibers at 5 and 14 dpi; 100–300 myofibers were analyzed from *n =* 6 mice per group. (**C**) Tibialis anterior (TA) muscle weight (normalized to body weight) from mice of the indicated genotype and treatment condition at 14 dpi. (**D**) Representative H&E and immunofluorescence images of muscle cross sections at 14 dpi. Sections were stained with antibodies targeting fiber-type-specific myosin heavy chains, including Myh7 (type I, purple), Myh2 (type IIa, red), Myh4 (type IIb, blue), Myh1 (type IIx, red), and Myh8 (neonatal, red), and myofiber boundaries (laminin, green). Scale bars: 50 μm. Statistical significance was assessed using 1-way ANOVA (**C**) or Kruskal-Wallis (**B**) test with adjustments for multiple comparisons. Box-and-whisker plots indicate median (horizontal line) and interquartile ranges (bounds of the boxes) from the indicated number of biological replicates; whiskers were plotted using Tukey’s method.

Loss of Mfn2 is predicted to have myriad cellular and organellar effects in regenerating myofibers. However, our above results with the PX-478 compound suggest that elevated HIF1α signaling represents a key feature governing regenerative defects in our animal model. To definitively test the relevance of excess HIF1α signaling in *mfn2^–/–^* regenerating myofibers, we made use of a conditional knockout allele to deplete HIF1α levels in MuSCs (Supplemental Figure 8A). Consistent with a previous report (39), *Hif1α* deletion alone in MuSCs did not impair regeneration of myofibers in response to injury, including the activation of MuSCs, and the formation and maturation of new myofibers (Figure 7, A–D, and Supplemental Figure 8, B and C). In the background of *Mfn2* deletion, *Hif1α* removal largely rescued myofiber maturation defects, including significant improvements in myofiber and muscle size, mitochondrial content, as well as robust differentiation into adult fiber types (Figure 7, A–D, and Supplemental Figure 8D). Thus, elevated HIF1α signaling is a key functional mechanism by which Mfn2 governs muscle regeneration.

**Figure 8:**
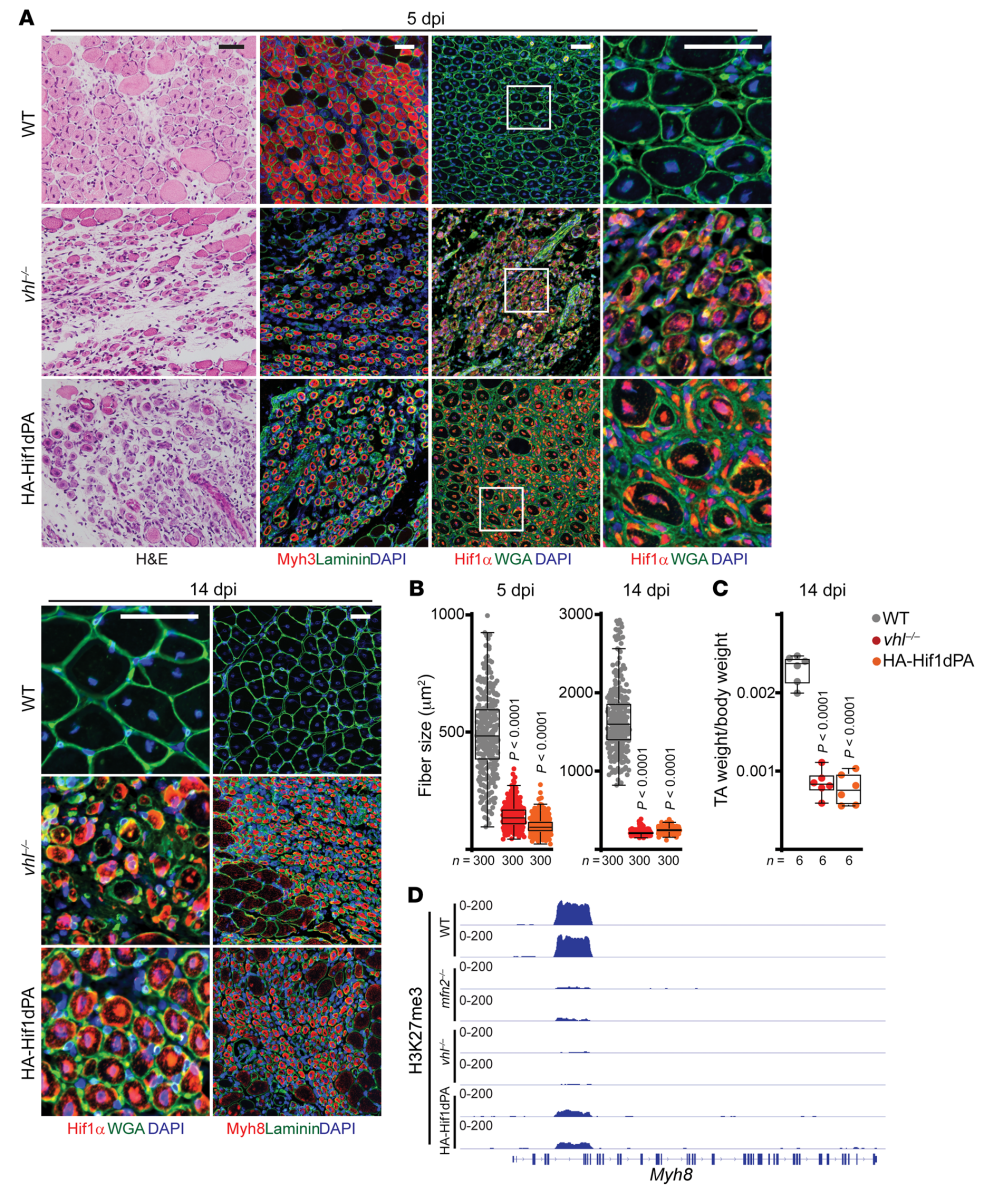
Increased HIF1α signaling inhibits maturation in regenerating myofibers. (**A**) Representative H&E and immunofluorescence images of muscle cross sections at 5 and 14 dpi. Sections were stained with antibodies targeting Myh3 (red), HIF1α (red), or Myh8 (red), as well as nuclei (DAPI, blue), and myofiber boundaries (wheat germ agglutinin [WGA, green] or laminin [green]). Scale bars: 50 μm. (**B**) Cross-sectional area of regenerating fibers from muscles at 5 and 14 dpi; 300 myofibers were analyzed from *n =* 6 mice per group. *P* values reflect comparison with wild-type group. (**C**) TA muscle weight (normalized to body weight) from mice of the indicated genotype and treatment condition at 14 dpi. *P* values reflect comparison with wild-type group. (**D**) Representative snapshots of H3K27me3 deposition at the *Myh8* gene in 14-dpi myofibers of the indicated genotype. For each ChIP-seq data set, 3 biological replicates were analyzed. Statistical significance was assessed using 1-way ANOVA (**C**) or Kruskal-Wallis (**B**) test with adjustments for multiple comparisons. Box-and-whisker plots indicate median (horizontal line) and interquartile ranges (bounds of the boxes) from the indicated number of biological replicates; whiskers were plotted using Tukey’s method.

These data suggest the possibility of a HIF1α-mediated regeneration checkpoint that regulates the transition between neonatal and adult fiber types. While loss of HIF1α has been previously studied in MuSCs (39), the effects of excess HIF1α in regenerating myofibers has yet to be examined to the best of our knowledge. We therefore made use of conditional alleles targeting HIF1α stability, combined with the *Pax7-Cre^ERT2^* driver. In these experiments, we prevented degradation of HIF1α by either conditional removal of VHL (the substrate recognition module for E3 ligase–mediated degradation of HIF1α), or conditional expression of a proline→alanine mutant of HIF1α (HA-HIF1dPA) that prevents recognition by VHL (Supplemental Figure 8, E and F) (48, 49). In both models, we observed significant increases in HIF1α levels after tamoxifen and during regeneration (Figure 8A). Both genetic models largely recapitulated key features of the *mfn2^–/–^* model, including normal activation of MuSCs, decreased fiber size and muscle weight, arrested fibers at the neonatal Myh8^+^ stage, and loss of H3K27me3 deposition at the *Myh8* locus (Figure 8, A–D, and Supplemental Figure 8, G and H). Thus, elevated HIF1α signaling during muscle regeneration is sufficient to inhibit myofiber maturation, including a specific blockade of the neonatal-to-adult fiber type transition.

### Neonatal Myh8^+^ fibers are characteristic in severe CNM.

**Figure 9:**
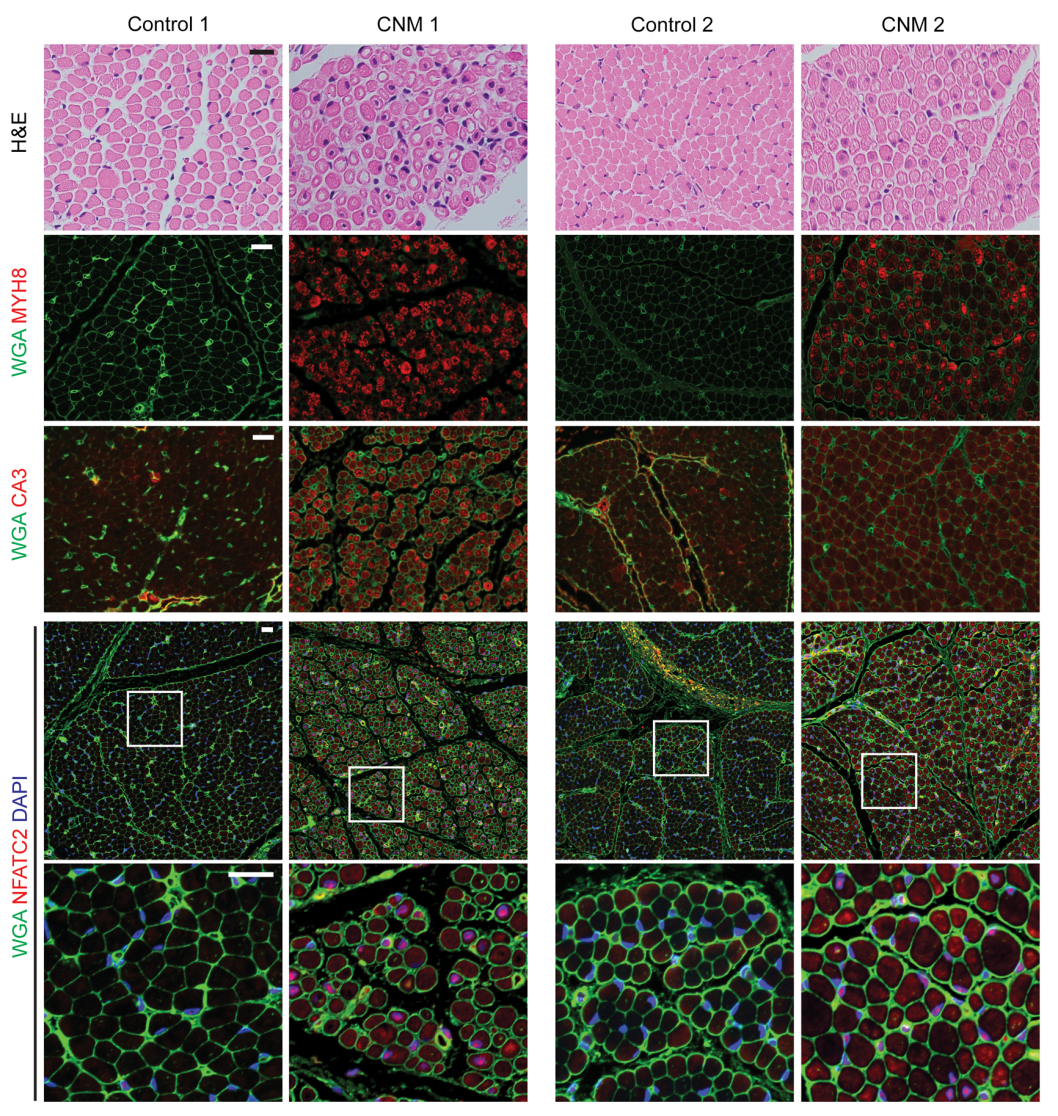
Myh8^+^ fibers are characteristic of severe centronuclear myopathy. Representative images of histology (H&E) and immunofluorescence for MYH8, CA3, and NFATC2 from a patient with infantile CNM due to a *Dnm2* mutation (CNM1) and an age- and sex-matched control (Control 1), and a patient with infantile CNM due to a *Mtm1* mutation (CNM2) and an age- and sex-matched control (Control 2). Myofiber boundaries were visualized with wheat germ agglutinin (WGA) staining, and nuclei were visualized with DAPI staining. Scale bars: 20 μm.

The histological deficits present in *mfn2^–/–^* regenerating myofibers are reminiscent of a CNM phenotype. CNM comprises a group of rare genetic muscle disorders with variable severity, ranging from life-threatening infantile presentations to milder adult-onset forms (50). Histological findings include centrally placed nuclei within muscle fibers, sometimes accompanied by perinuclear mitochondria. A number of genes have been implicated in CNM, including *Mtm1*, *Dnm2*, *Bin1*, and *Ryr1*; however, the precise disease pathophysiology is still under investigation. We therefore investigated myofiber fate and HIF1α status in muscle biopsies from genetically confirmed CNM patients and age- and sex-matched controls (Supplemental Table 5). Two patients with infantile CNM displayed a substantial number of central nuclei myofibers with perinuclear mitochondrial localization (Figure 9 and Supplemental Figure 9A). Interestingly, CNM myofibers stained strongly positive for the neonatal MYH8 marker (Figure 9). We investigated HIF1α status in the severely affected patients, making use of staining for carbonic anhydrase 3 (CA3), a HIF1α target and marker of hypoxia in clinical specimens (51, 52). From this analysis, we observed elevated CA3 levels in the severe CNM samples as compared with controls (Figure 9). We also observed increased nuclear localization of NFATC2 in CNM patients, particularly the patient with a *DNM2* mutation (CNM1; Figure 9). Thus, our findings suggest that affected fibers in severe CNM mimic results from our *Mfn2*-mutant animal models, including a maturation arrest at the neonatal MyH8 stage and elevated HIF1α activity, although CNM disease is not associated with MFN2 deficiencies (Supplemental Figure 9A).

### Regenerating myofibers following ischemic injury engage a HIF1α-dependent checkpoint at the neonatal-adult fate transition.

**Figure 10:**
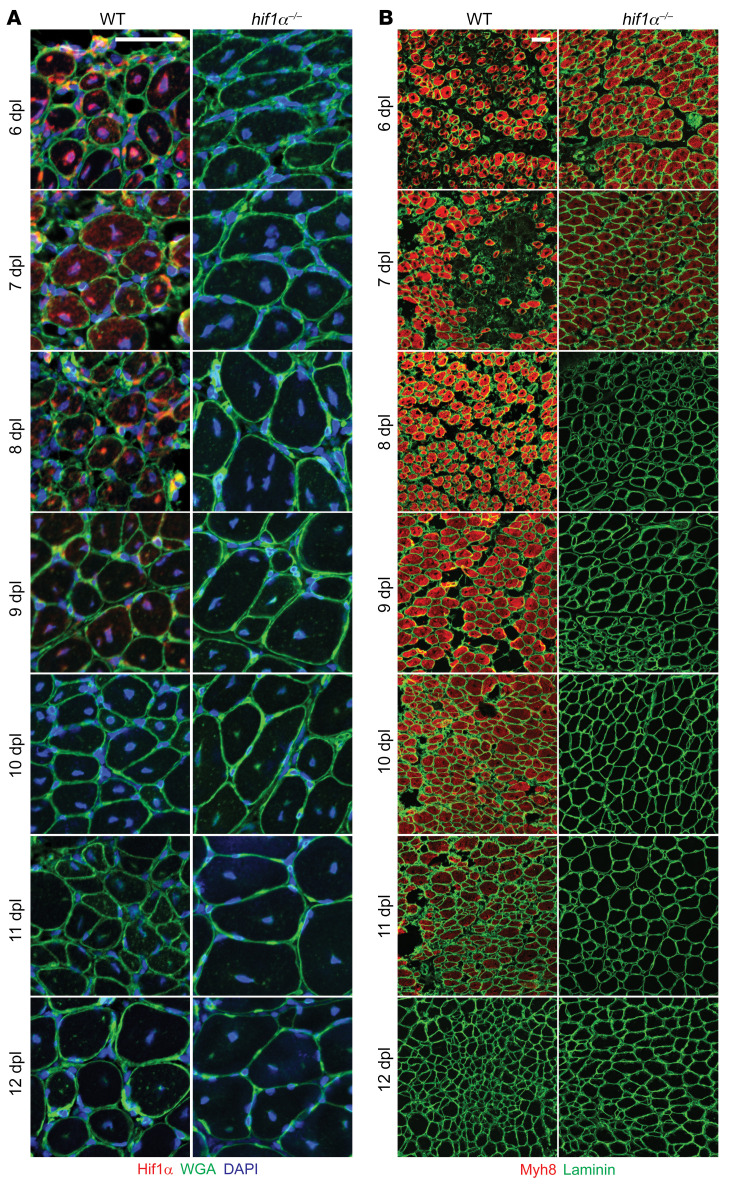
Regenerating myofibers pause at the neonatal-adult transition in response to ischemic injury. (**A**) Representative immunofluorescence images of muscle cross sections from wild-type and *hif1α^–/–^* mice at the indicated time points (days post ligation, dpl). Muscle cross sections were stained with antibodies targeting HIF1α (red). Nuclei were visualized with DAPI, and myofiber boundaries were visualized with laminin staining (green) or wheat germ agglutinin (WGA, green). Scale bar: 50 μm. (**B**) Same as **A**, except cross sections were stained with antibodies targeting Myh8 (neonatal myosin heavy chain, red). Myofiber boundaries were visualized with laminin staining (green). Scale bar: 50 μm.

**Figure 11:**
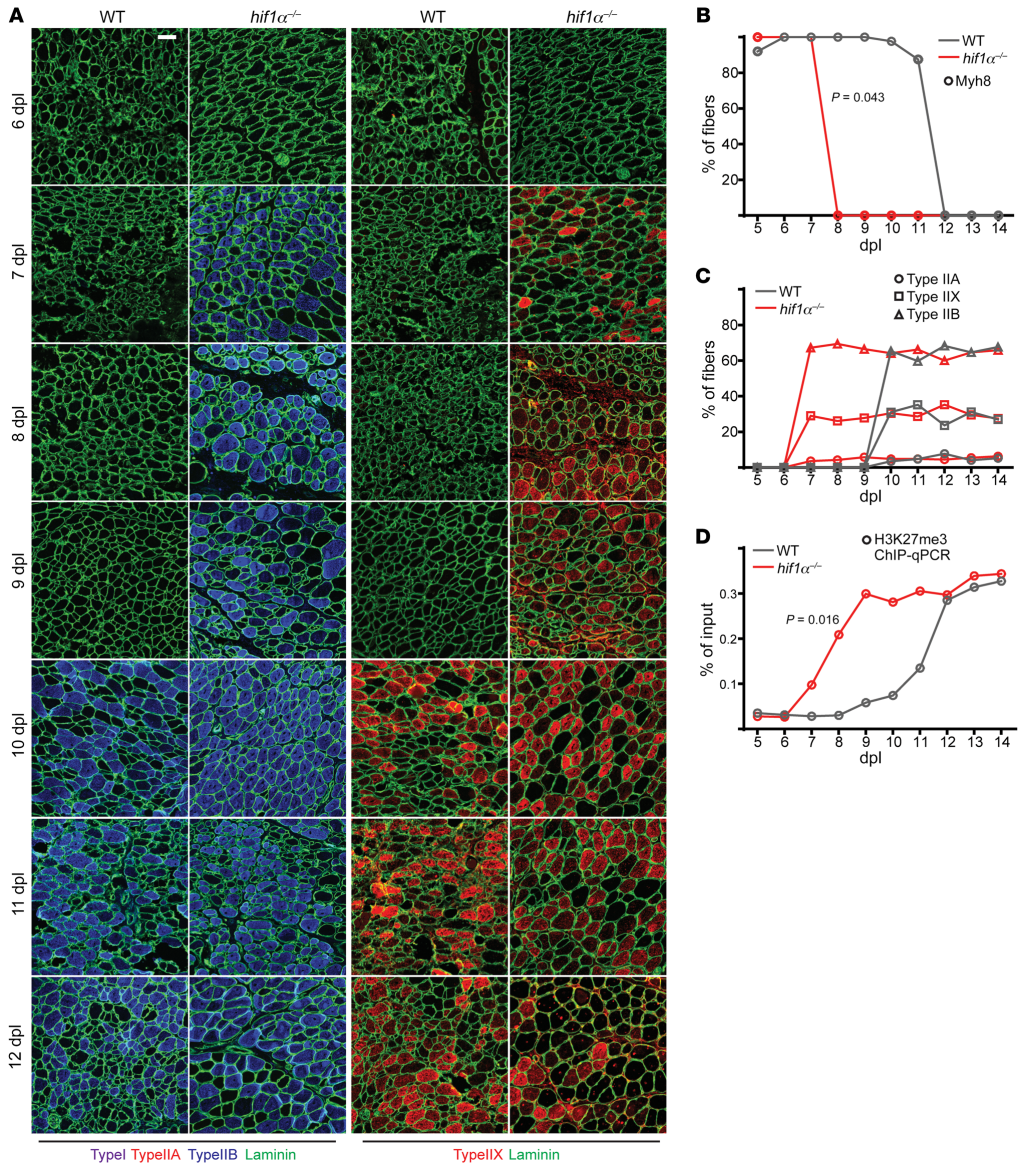
*Hif1α* deletion accelerates myofiber maturation during muscle regeneration in response to ischemic injury. (**A**) Representative immunofluorescence images of muscle cross sections from wild-type and *hif1α^–/–^* mice at the indicated time points (days post ligation, dpl). Muscle cross sections were stained with antibodies targeting fiber-type-specific myosin heavy chains, including Myh7 (type I, purple), Myh2 (type IIa, red), Myh4 (type IIb, blue), and Myh1 (type IIx, red). Myofiber boundaries were visualized with laminin staining (green). Scale bar: 50 μm. (**B**) Quantification of the percentage of regenerating myofibers positive for Myh8 staining in the indicate genotypes. (**C**) Quantification of the percentage of regenerative myofibers of the indicated adult fiber type, in the indicated genotypes. (**D**) Quantification of enrichment (% of input) from H3K27me3 ChIP-qPCR experiments targeting the *Myh8* gene. Experiments were performed in regenerating myofibers from animals of the indicated genotype and time point. *n =* 1 animal per genotype per time point. Statistical significance was assessed using 2-way ANOVA (**B** and **D**).

The above results reveal that genetic modifications associated with excess HIF1α levels are sufficient to arrest regenerating myofibers at the neonatal (Myh8^+^) stage following chemically induced injury. In principle, arrested myofibers may delay muscle recovery by inhibiting tissue maturation and growth. We therefore investigated whether wild-type animals engage a similar arrest during recovery from severe muscle injury. We implemented an ischemic injury model, making use of a femoral artery ligation protocol that robustly limits blood flow to the affect limb and is associated with severe muscle injury (53, 54). We assessed wild-type animals at 5 to 14 days post ligation (dpl), and noted that HIF1α levels declined at 7–9 dpl in the TA muscle (Figure 10A and Supplemental Figure 10A), which correlates with the timing of reperfusion of the limb by peripheral arteries (based on previous studies; ref. 54). Wild-type regenerating myofibers remained arrested in the neonatal Myh8^+^ state until day 9, at which point Myh8 levels began declining and were undetectable by day 12 (Figure 10B and Supplemental Figure 10A). Concomitantly, we observed an increasing deposition of H3K27me3 marks at the *Myh8* locus starting on day 9 (Figure 11D), as well as the appearance and growth of adult fiber types starting on day 10 (Figure 11A and Supplemental Figure 10A). Thus, in response to ischemic injury, regenerating myofibers remain arrested in a Myh8^+^ state for several days, and transition from a neonatal to adult fate coincident with reperfusion, lowering of HIF1α levels, and H3K27me3 deposition at the *Myh8* locus.

To assess the role of HIF1α in this process, we performed femoral artery ligations in the setting of conditional removal of *Hif1α*. In these animals, myofiber maturation was significantly accelerated (Figure 11, B and C). In particular, Myh8^+^ fibers had completely differentiated by day 8, approximately 4 days earlier as compared with wild-type animals (Figure 10B, Figure 11B, and Supplemental Figure 10A). These events correlated with an earlier deposition of H3K27me3 marks at the *Myh8* locus, and an earlier appearance and growth of adult fiber types (Figure 11, A, C, and D). Thus, regenerating myofibers arrest at a Myh8^+^ state in the setting of severe injury in a HIF1α-dependent manner. In the setting of *Hif1α* removal, this arrest is bypassed, resulting in accelerated growth and maturation of muscle tissue.

## Discussion

The ability of HIF1α to regulate fate specification of regenerating myofibers constitutes a new physiological role for its already versatile signaling pathway. In regenerating muscle, HIF1α activity regulates myofiber fate specification and epigenetic control of the neonatal *Myh8* locus, where repressive H3K27me3 marks appear necessary to suppress expression of developmental myosins and allow fibers to adopt adult fates. These data support a model in which muscle regeneration contains a checkpoint that prevents adult fiber type specification in the presence of HIF1α signaling. Importantly, inhibition of HIF1α allows myofibers to pass this checkpoint, thereby accelerating the regeneration process. We note that a limitation of our study is that our experiments exclusively focused on the TA muscle of mice, which predominantly consists of fast-twitch (type IIX and IIB) fibers. It is possible that other muscles with alternative fiber type compositions, including human muscles, may respond differently during the regenerative process.

The involvement of HIF1α suggests that ischemia and reperfusion of the injured area play important roles in regulating myofiber specification during muscle repair. Indeed, a large number of studies have previously assessed the role of both hypoxia and hyperoxia in MuSC activation, in both in vitro and in vivo contexts (55). Although some of these studies have provided mixed results, hyperbaric oxygen therapy has been shown in animal models to increase the size of myofibers following muscle injury, and is commonly used in athletes to promote muscle recovery (56–58). The precise mechanisms relating hyperoxia to muscle regeneration are unknown, but have largely focused on proliferation of activated MuSCs. A previous study (39) indicated that removal of HIF1α in MuSCs allows for an increased number of ASCs that promoted increased size of regenerated myofibers. Our results complement these findings by showing that loss of HIF1α also promotes muscle regeneration by accelerating transition of myofibers into an adult fate.

Our data add to the proposed techniques to enhance muscle regeneration by suggesting a therapeutic intervention targeted to the latter stages of muscle repair, when newly developed myofibers are growing and transitioning into adult fiber types. Previous work on enhancing muscle growth during recovery has largely focused on nutrient supplementation, physical therapy, and mechanical scaffolds (59). We show above that there exists a HIF1α-dependent pause at the neonatal-adult transition, which is targetable and offers an opportunity to accelerate muscle repair through the use of orally available HIF1α or KDM6 inhibitors. HIF1α loss in skeletal muscle is well tolerated in animal studies (60), indicating that bypassing this checkpoint does not significantly impair tissue health. The KDM6 inhibitor used here (GSK-J4) has been reported to inhibit the expression of myogenin during early stages of muscle regeneration (61), suggesting that the beneficial effects of demethylase inhibition are restricted to the latter stage of muscle regeneration. In future work, it will be interesting to test whether these inhibitors synergize with therapies targeted to earlier stages of muscle regeneration (MuSC activation, immune cell clearance). In addition, developing muscle fibers in the embryonic and neonatal stages go through a parallel fate specification pattern (9), and we found that pediatric CNM patients with severe disease exhibit histological and fate specification characteristics similar to our *mfn2^–/–^* regenerating myofibers. It will be interesting to explore whether a similar HIF1α checkpoint regulates the timing of adult fiber specification and growth during development, and impacts the development of CNM pathology.

These results also highlight a role for mitochondria in MuSC function beyond their function as ATP generators. Here, we find that a key role for Mfn2 in regenerating myofibers relates to its ability to negatively regulate HIF1α signaling. Removal of Mfn2 alone is largely dispensable in adult muscle fibers (23, 62, 63), but is predicted to have myriad cellular and organellar effects. Our results instead highlight a context-specific requirement for Mfn2 in regenerating myofibers that is functionally mediated by alterations in HIF1α signaling. Interestingly, we observed similar phenotypes with overexpression of the CMT2A disease–associated *Mfn2^T105M^* allele, suggesting that delayed myofiber regeneration may contribute to the disease pathophysiology in associated patients. Muscle biopsies are not routinely performed on CMT2A patients, and thus it will be important to assess in a future prospective study whether these patients suffer from impaired tissue regeneration.

## Methods

Detailed method information, including information on statistical analyses, is included in Supplemental Methods.

### Data and material availability.

Raw and processed data for RNA-seq and ChIP-seq experiments are available at the NCBI Gene Expression Omnibus (GEO GSE185106). Other data and materials are provided within the manuscript and supplementary materials, or available upon reasonable request.

### Study approval.

All animal studies were approved by the University of Texas Southwestern Medical Center Institutional Animal Care & Use Committee. Human studies were approved as a retrospective study on archived excess patient tissue by the University of Texas Southwestern Medical Center Institutional Review Board.

## Author contributions

XW conceptualized the study and contributed to methodology, validation, formal analysis, carrying out the investigation, data curation, writing the original draft and reviewing and editing the manuscript, and data visualization. YJ contributed to formal analysis, carrying out the investigation, and data curation. JZ contributed to formal analysis and carrying out the investigation. NPL, CJM, SDS, and SSKV contributed to carrying out the investigation. JX and CC provided critical resources and contributed to carrying out the investigation and reviewing and editing the manuscript. PM conceptualized the study, provided critical resources, and contributed to methodology, formal analysis, data curation, writing the original draft and reviewing and editing the manuscript, data visualization, supervision, and acquired funding.

## Supplementary Material

Supplemental data

Supplemental table 1

Supplemental table 2

Supplemental table 3

Supplemental table 4

Supplemental table 5

## Version 1 09/20/2022

In-Press Preview

## Version 2 12/01/2022

Electronic publication
