# Supplementary material for: A mitofusin 2/HIF1α axis sets a maturation checkpoint in regenerating skeletal muscle
Source: J Clin Invest. 2022 Dec 1;132(23):e161638. doi: 10.1172/JCI161638 (PMC9711883; doi:10.1172/JCI161638)
Supplement: Supplemental data [file jci-132-161638-s059.pdf]

Supplemental Materials for

A Mitofusin 2 – Hif1α axis sets a maturation checkpoint in regenerating skeletal muscle.

Xun Wang, Yuemeng Jia, Jiawei Zhao, Nicholas P. Lesner, Cameron J. Menezes, Spencer D. Shelton, Siva Sai Krishna Venigalla, Jian Xu, Chunyu Cai, Prashant Mishra

Supplemental Methods

Captions for Supplemental Tables S1 to S5

Supplemental Figures S1 to S12

Supplemental Methods

Mice

Throughout this study, all indicated genotypes refer to animals with conditional (floxed, 'f') alleles, targeted to the muscle satellite cell (MuSC) population using the *Pax7-Cre*^{ERT2} allele. *Mfn1*$^{f/f}$ (gift from David Chan), *Mfn2*$^{f/f}$ (gift from David Chan), *Rosa-STOP*$^{f/f}$-*Mfn2*^{T105M} (strain 025322), *Rosa-STOP*$^{f/f}$-*HA-HIF1dPA* (strain 009673), *Hif1*$\alpha^{f/f}$ (strain 007561), *Vhl*$^{f/f}$ (strain 012933), *Pax7-Cre*^{ERT2} (strain 012476), and *mito-Dendra2*$^{f/f}$ (strain 018385) were purchased from The Jackson Laboratory.

All mice were maintained on C57BL6 backgrounds, and conditional alleles were maintained in a homozygous state, and combined with Cre driver alleles by breeding. For *mfn2*$^{f/f}$ animals, we isolated a recombined chromosome with *Pax7-Cre*^{ERT2} as previously described (10). Both male and female mice were used in all experiments; if sex specific differences were not present, male and female mice were analyzed together. All mice were housed in the Animal Resource Center at the University of Texas Southwestern Medical Center under a 12 hour light-dark cycle and were fed ad libitum. All animal protocols were approved by the University of Texas Southwestern Institutional Animal Care and Use Committee (protocol 2015-101323).

Mouse injections

For tamoxifen (Cayman Chemical, #132585) injection of *Pax7-Cre*^{ERT2} mice (Figure 1A, 2C), tamoxifen was dissolved in corn oil (Sigma-Aldrich, #C8267), and 75 mg/kg body mass was administered by intraperitoneal injection to 6-8-week-old mice, once per day

for 5 consecutive days. Mice were euthanized and tissue harvested at various timepoints after tamoxifen administration as indicated.

For PX-478 (MedChem Express, #HY-10231) administration, starting at 14 days post injury, mice were treated with PX-478 (or vehicle, PBS) by intraperitoneal injection at 100 mg/kg body mass every other day for 14 days. Mice were then euthanized at 28 days post injury and tissue was harvested for analysis.

For GSK-J4 (Cayman Chemical, #12073) administration, starting at 14 days post injury, mice were treated with GSK-J4 (or vehicle, DMSO) by intraperitoneal injection at 75 mg/kg body mass every day for 14 days, based on a previous protocol (61). Mice were then euthanized at 28 days post injury and tissue was harvested for analysis.

Muscle injury experiments

For barium chloride ($BaCl_2$, Alfa Aesar, #0361-37-2) injury experiments, 6-8 week old mice were pretreated with tamoxifen as above, and a muscular $BaCl_2$ injury was administered based on previous protocols (64). Briefly, mice were anesthetized with isoflurane, and tibialis anterior (TA) muscles were directly injected with 50 μl of 1.2 % $BaCl_2$ (in sterile saline) using a sterile 29-gauge needle. Postoperative analgesia (meloxicam, 2 mg/kg/24 h, Sigma-Aldrich) was administered subcutaneously once per day for two days. Mice were euthanized and TA tissue harvested on the indicated days post injury.

For femoral artery ligation experiments, 6-8 week old mice were pretreated with tamoxifen as above. Femoral artery ligation was performed as previously described (53). Briefly, mice were anesthesized with isoflurane, followed by a surgical incision overlying the

medial left thigh muscle. The femoral artery was isolated and dissected away from the femoral vein and nerve, and ligated with 7-0 suture. Postoperative analgesia (meloxicam, 2 mg/kg/24 h, Sigma-Aldrich) was administered subcutaneously once per day for two days. Mice were euthanized and TA tissue harvested on the indicated days post-ligation.

Isolation of regenerating muscle for analysis

Following anesthesia under isoflurane, the tibialis anterior muscles were rapidly dissected, and immediately frozen in liquid nitrogen cooled 2-methylbutane. For histology analysis, frozen muscles were embedded in O.C.T compound (Fisher Scientific, # 23-730-571). 10 μM sections were cut on a cryostat (Leica CM3050S). For H&E staining, slides were prepared following the protocol from the TREAT-NMD website (http://www.treat-nmd.eu/downloads/file/sops/cmd/MDC1A_M.1.2.004.pdf). Sections were imaged using an Olympus IX83 microscope and analyzed with Image J software (NIH) to calculating myofiber numbers and size. For other analyses (western blot, ChIP-seq, ChIP-qPCR, qRT-PCR, qPCR), fresh muscles were imaged on a Nikon SMZ18 stereomicroscope and regenerating Dendra^{+} sections were dissected and flash frozen at -80°C for further analysis.

Metabolite Mass Spectrometry Analysis

Frozen muscles were pulverized on liquid nitrogen, and metabolites extracted with 80% acetonitrile and centrifuged at 17,000xg at 4 °C. The metabolite containing supernatant was injected onto a HILIC column (Millipore ZIC-pHILIC, 5 μm, 2.1 × 150 mm). Chromatographic separation was achieved using 10 mM ammonium acetate, pH 9.8

(solvent A) and acetonitrile (solvent B) with a constant flow rate of 0.25 mL min^{-1}. The column was equilibrated with 90% solvent B. The gradient was as follows: 0-15 min linear ramp from 90% B to 30% B; 15-18 min isocratic flow of 30% B; 18-19 min linear ramp from 30% to 90% B; 19-27 min column regeneration with isocratic flow of 90% B. Metabolites were detected with a QExactive HF-X hybrid quadrupole orbitrap high-resolution mass spectrometer (Thermo Scientific) coupled to a Vanquish UHPLC. Data was acquired using HRMS full scan (precursor ion only) switching between polarities. Data was integrated and analyzed using El-MAVEN (Elucidata) (65).

Human Subjects

Human muscle biopsy specimens were collected from archived patient frozen muscle biopsy tissue stored at -84°C at the UT Southwestern Neuropathology laboratory. The tissue usage was approved as a retrospective study on archived excess patient tissue by the local ethics committee (STU 012016-082), thereby waiving the need for further consent. Only de-identified relevant patient information was provided. Control human muscle tissue were from age and gender matched patients who underwent muscle biopsy for various reasons and had normal muscle histology and muscle enzyme histochemical studies.

Immunofluorescence protocols

For mouse muscle sections, freshly frozen 10 μm sections (prepared as above) were fixed in formalin at room temperature for 5 min and then blocked with blocking buffer ((0.25% Triton X-100 (Sigma-Aldrich, #X100) and 10% goat serum (GIBCO, # 16210064))

in PBS at room temperature for 1 hour. Sections were incubated with primary antibodies diluted in blocking buffer at 4°C overnight. On the second day, sections were washed with PBS and then incubated with secondary antibodies diluted in blocking buffer at room temperature for 1 hour. Sections were stained with DAPI diluted in PBS, and then washed with PBS and mounted with fluoro-gel mounting medium (Electron Microscopy Sciences, #1798510).

For human muscle sections, slides were deparaffinized with xylene and rehydrated with a graded series of ethanol (100%, 90%, 80%, 70%, 50%, 30% and distilled water). Antigen retrieval was performed with sodium citrate buffer (10 mM pH 6). Sections were blocked with blocking buffer at room temperature for 1 hour. Sections were incubated with primary antibodies diluted in blocking buffer at 4°C overnight. On the second day, sections were washed with PBS and then incubated with secondary antibodies diluted in blocking buffer at room temperature for 1 hour. Sections were stained with DAPI diluted in PBS, and then washed with PBS and mounted with fluoro-gel mounting medium.

For myotube staining, myotube cultures were fixed in formalin at room temperature for 5 min and then blocked with blocking buffer at room temperature for 1 hour. Cells were incubated with primary antibodies diluted in blocking buffer at 4°C overnight. On the second day, cells were washed with PBS and then incubated with secondary antibodies diluted in blocking buffer at room temperature for 1 hour. Cells were stained with DAPI, diluted in PBS, and then washed with PBS and mounted with fluoro-gel mounting medium.

Slides were imaged using a Zeiss LSM780 Inverted confocal microscope and analyzed using ImageJ software. The following antibodies were used: Myh3 (BF-45), Myogenin

(F5D), Myh (MF20), Myhc type I (BA-D5), Myhc type IIa (SC-71), Myhc type IIb (BF-F3), Myhc type IIx (6H1), Myh8 (N3.36), CD31 (2H8) (all from Developmental Studies Hybridoma Bank, 2 μg/ml), MyoD (Santa Cruz Biotechnology, #sc-32758, 1:500), Laminin (Sigma-Aldrich, #L9393, 1:500), Hif-1α (Novus Biologicals, #NB100-479, 1:500), NFATc2 (Thermo Fisher, #MA1-025, 1:500), Mfn2 (Proteintech, #12186-1-AP, 1:500), Alexa Fluor 488 WGA (Thermo Fisher, #W11261, 5 µg/mL), DyLight 405 Goat Anti-mouse IgM (Jackson ImmunoResearch, #115-475-075, 1:500), Alexa Fluor 647 goat anti-Armenian hamster (Jackson ImmunoResearch, #127-605-160, 1:500), Alexa Fluor 594 goat anti-rabbit (#A11012), Alexa Fluor 594 goat anti-mouse IgM (#A21044), Alexa Fluor 594 goat anti-mouse IgG2b (#A21145), Alexa Fluor 594 goat anti-mouse IgG1 (#A21125), Alexa Fluor 488 goat anti-rabbit IgG(H+L) (#A11034), and Alexa Fluor 647 goat anti-mouse IgG2b (#A21242) (all from Invitrogen, 1:500).

<u>MuSCs isolation via Fluorescence-activated cell sorting (FACS)</u>

Murine MuSCs were isolated follow a previously reported protocol (66). Following carbon dioxide asphyxiation and cervical dislocation, skeletal muscle was rapidly dissected and sequential digested with Collagenase II (1 hr) and Dispase (30 minutes) at 37°C. Mononucleated cells were collected through a 70 μm cell strainer, and suspended in HBSS with 2% horse serum (GIBCO, #16050114). Cells were then incubated with the following antibodies on a rotator at 4°C for 30 min: APC-conjugated anti-mouse CD31 (BioLegend, clone MEC13.3, #102510, 1:100), APC-conjugated anti-mouse CD45 (BioLegend, clone 30-F11, #103112, 1:100), PerCP-Cy5.5-conjugated anti-mouse Sca-1 (Invitrogen, Clone D7, #45598182, 1:100), Biotin-conjugated anti-mouse CD34

(Invitrogen, clone RAM34, #13034181, 1:100). After incubation, cells were washed twice, and then incubated with PE/Cy7-conjugated streptavidin (BioLegend, 1:100, #405206) on a rotator at 4 °C for 20 min. Cells were washed twice, and then suspended with 2% horse serum in HBSS with DAPI. Quiescent MuSCs were identified as the CD34$^+$, CD31$^-$, CD45$^-$, DAPI$^-$ and Sca-1$^-$. Purity was confirmed by immunofluorescence staining for Pax7 (ab528428, Developmental Studies Hybridoma Bank). A representative gating strategy is provided in Figure S11A.

For quiescent and activated MuSCs after injury, mononucleated cells from mito-Dendra2 mice were resuspended with 2% horse serum in HBSS with DAPI. Quiescent MuSCs were identified as the DAPI$^-$, GFP$^+$ and CD34$^+$, and activated MuSCs were identified as DAPI$^-$,GFP$^+$ and CD34$^-$. A representative gating strategy is provided in Figure S11B. All sorting was performed at the Moody Foundation Flow Cytometry Facility on a FACS Aria flow cytometer (BD Biosciences). Data was analyzed by FACSDiva (BD Biosciences) or FlowJo (Version10.6.1) software.

<u>In vitro MuSC proliferation and fusion assay</u>

To measure MuSC proliferation, 10,000 freshly isolated MuSCs were plated in each well of a 12-well plate with growth medium (Ham's F-10 (HyClone, SH30025.01)) supplemented with 10% horse serum, 1% penicillin/streptomycin and 2.5 ng/ml basic fibroblast growth factor (Preprotech, 100-18B). Cell numbers per well were counted each day using a Celigo imaging cytometer (Nexcelom Bioscience, 5.1.0.0).

To measure MuSC fusion, 100,000 freshly isolated MuSCs were plated in an eight-well chamber slide (Thermo Fisher Scientific, 177445) with growth medium (Ham's F-10

(HyClone, SH30025.01)) supplemented with 10% horse serum, 1% penicillin/streptomycin and 2.5 ng/ml basic fibroblast growth factor (Preprotech, 100-18B) for 24 hour. The media was then switched to differentiation medium (DMEM with 2% horse serum and 1% penicillin/ streptomycin) for 4 days. Cells were fixed and stained with antibodies against myosin heavy chains (Myh (MF20, Developmental Studies Hybridoma Bank), and Myh8 (N3,36, Developmental Studies Hybridoma bank) and DAPI. The fusion index was calculated as the fraction of total nuclei in myosin-positive myotubes.

Mitochondrial reactive oxygen species (ROS) and mitochondrial membrane potential (ΔΨm) analysis

Mitochondrial ROS and mitochondrial membrane potential were measured in isolated MuSCs at 5 days after the first dose of tamoxifen as previously described (67). For mitoROS measurements, MuSCs were resuspended in HBSS with 5 μM final concentration mitoSOX (mitochondrial superoxide levels, Thermo Fisher, M36008) and then incubated at 37°C for 30 minutes. After incubation, cells were washed and resuspended in HBSS with DAPI, followed by immediate FACS analysis.

For mitochondrial ΔΨm measurements, isolated MuSCs were resuspended in mitochondrial assay buffer with the indicated mitochondrial substrates and inhibitors (see below for recipes). Cell suspensions were incubated at 37 °C for 30 min. After incubation, cells were washed and then suspended in HBSS with DAPI, followed by immediate FACS analysis.

The mitochondrial assay buffer contained 220 mM mannitol, 70 mM sucrose, 10 mM KH_2PO_4, 5 mM $MgCl_2$, 2 mM HEPES and 1 mM EGTA, pH 7.4, supplemented with fresh

TMRE (Invitrogen, T669; final concentration, 150 nM) and fresh PMP reagent (Agilent, 102504-100; final concentration, 3 nM). Pyruvate/malate buffer contained mitochondrial assay buffer supplemented with 10 mM pyruvate and 5 mM malate, pH 7.4. Inhibitor concentrations were 5 μM for CCCP and 5 μM for rotenone.

Extracellular flux (Seahorse) assay

Freshly isolated MuSCs were plated in Seahorse XFe96 cell culture plates overnight with growth medium (Ham's F-10 (HyClone, SH30025.01)) supplemented with 10% horse serum, 1% penicillin/streptomycin and 2.5 ng/ml basic fibroblast growth factor (Preprotech, 100-18B). The following day, cells were washed twice with 200 μl per well assay medium (DMEM (Sigma-Aldrich, D5030) with 10 mM glucose, 2 mM L-glutamine, 1 mM sodium pyruvate and 1% penicillin/streptomycin), and 150 μl assay medium was added to each well after the second wash. Cells were transferred to a 37°C, CO_2-free incubator for 1 hour. Oxygen consumption measurements were performed in a Seahorse XFe96 instrument using a 3 minute mix, 3 minute measure cycle with three measurements recorded at baseline and after injection of each compound. The following inhibitors were sequentially used at the indicated final concentrations: 2 μM oligomycin, 3 μM CCCP (carbonyl cyanide m-chlorophenyl hydrazone) and 3 μM antimycin A. Data collection was performed with WAVE (v.2.4.1.1) software. At the completion of the experiment, cells were fixed with formalin, stained with DAPI, and cell counts were measured per well using a Celigo imaging cytometer (Nexcelom Bioscience, 5.1.0.0). Mitochondrial OCR (oxygen consumption rates) were calculated as basal (pre-oligomycin) OCR – baseline (post-antimycin) OCR. Maximal OCR was calculated as

CCCP-stimulated OCR – baseline (post-antimycin) OCR. OCR values were normalized by the cell count per well.

RNA isolation and sequencing

Total RNA was purified from FACS-isolated MuSCs using the RNeasy Micro Kit (QIAGEN # 74004) according to manufacturer's instructions. Library preparation was performed using the SMARTer stranded pico input total RNA-seq kit (Takara, #634411) following manufacturer instructions. Next generation sequencing was performed using an Illumina NextSeq 500 by the Children's Research Institute's Sequencing Facility at UT Southwestern Medical Center. RNA-seq analysis was performed on BICF RNASeq Analysis Workflow (ssh://git@git.biohpc.swmed.edu/BICF/Astrocyte/rnaseq.git) provided by the UTSouthwestern Bioinformatics Core Facility. Hif-1α pathway enrichment analysis was performed using Gene set enrichment analysis (GSEA) (68, 69). Gene ontology analysis was performed using DAVID (https://david-d.ncifcrf.gov/home.jsp).

Chromatin Immunoprecipitation Sequencing (ChIP-Seq) and ChIP-qPCR

ChIP-Seq was performed as described previously (70, 71). Isolated MuSCs or minced myofibers were crosslinked in 1% final concentration formaldehyde in PBS for 10 min at room temperature, and then quenched using 125 mM final concentration glycine for 10 min at room temperature. For myofibers samples, the tissue was then dounced in cold PBS. Following filtering with a 40 μm cell strainer, the tissue was centrifuged at 2000 rpm for 5 min, and then washed twice with cold PBS. Chromatin was sonicated to around 500 bp in buffer 0 (10 mM Tris-HCl, 1 mM EDTA, 0.1% sodium deoxycholate, 0.1% SDS, 1%

Triton X-100, 0.25% sarkosyl, pH 8.0). Sonicated chromatin was incubated with primary antibodies at 4 °C overnight. On the second day, protein A/G Dynabeads (Thermo Fisher, #PI26162) were added to the ChIP reactions and incubated for 3 hours at 4°C. Dynabeads were separated and washed twice with 1 ml of buffer 0, twice with 1 ml of buffer 0.3 (buffer 0 with 0.3 M NaCl), twice with 1 ml of LiCl buffer (10 mM Tris-HCl, 1 mM EDTA, 0.5% sodium deoxycholate, 0.5% NP-40, 250 mM LiCl, pH 8.0), and twice with 1 ml of TE buffer (10 mM Tris-HCl, 1 mM EDTA, pH 8.0). The chromatin was then eluted in SDS buffer (1% SDS, 10 mM EDTA, 50 mM Tris-HCl, pH 8.0) followed by reverse crosslinking at 65°C overnight. Pulldown DNA was incubated at 37°C with RNaseA (10 mg/ml) and protease K (0.2 mg/ml) for 2 hrs to remove residual RNA and protein, and then purified using QIAquick Spin Columns (Qiagen, #28004). ChIP-seq experiments were performed using previously validated antibodies: MyoD (Santa Cruz Biotechnology, #sc-32758X), Myogenin (Developmental Studies Hybridoma Bank, #F5D), H3K9me3 (Abcam, #ab8898), H3K27me3 (Abcam, #ab6002), NFATc2 (Thermo Fisher, #MA1-025). ChIP-seq libraries were prepared using NEBNext ChIP-Seq Library Prep Master Mix Set for Illumina Kit (New England Biolabs, #E7645S). Next generation sequencing was performed using an Illumina NextSeq 500 (Children's Research Institute's Sequencing Facility at UT Southwestern Medical Center). For ChIP-seq experiments, we analyzed three independent biological replicates in each group; except for NFATC2 binding, where two independent biological replicates were analyzed in each group. Reads were aligned against the reference genome mm10 with BWA (version 0.7.5) (72). Peak calling was performed using MACS14 (version 1.4.2) using option -nomodel and p-value cutoff of 1e-5 (73). The PcG target geneset (Table S2) was taken from (35). Differential peaks were

identified using DiffBind (version 3.2.2) in R with a peak size of 250bp and DBA_NORM_TMM normalization. For MyoD and MyoG ChIP-seq experiments, bigwig files were generated from BAM files using bamCoverage from deeptools (version 3.5.0) with 10 bp bin size, smoothLength of 100 bp, extendReads of 200 bp and a scaled factor calculated from total reads. For H3K9me3 and H3K27me3 ChIP-seq experiments, bigwig files were generated from BAM files using bamCoverage from deeptools (version 3.5.0) with 10 bp bin size, smoothLength of 100 bp, extendReads of 200 bp and a scaled factor. The scale factor for each sample was calculated based on normalization factor from dba.normalize in DiffBind (version 3.2.2) using normalization method DBA_NORM_TMM. To generate the peak heatmap graphs on H3K9me3 and H3K27me3, we generated merged fastq files by combining the three replicates from the same condition, and then ran the analysis as described above to generate a merged bigwig file. The peak heatmaps were plotted using deeptools (3.5.0) with default parameters. k-means clustering was performed with deeptool (version 3.5.0) with option -kmeans on normalized merged bigwig files.

For motif analysis (Figure S12A-C): To generate fasta files, BED files of H3K27me3 and H3K9me3 ChIP-seq containing peak information of each genotype (WT, Mfn2, Vhl, Hif1) were combined and merged by bedtools merge command. The fasta files were then generated by extracting the sequence annotated in the BED files with mm10 genome as the reference. The prepared fasta files were then used for motif discovery by the meme-chip software in MEME suite package.

To generate footprinting/enrichment plots (Figure S3F, S12D), the BigWig files of each group were first merged by taking the mean and outputted as averaged Wiggled files

using wiggletools software (“mean” as the operator). The wiggled files were then converted back to BigWig files using the WigToBigwig command of UCSC tools. To get transcription factor motif specific BED files, the whole genome level BED files were generated using scanmotifgenomewide.pl command from the Homer software package. For Myod and Myog, the Homer compatible motif files were downloaded directly from the Homer motif library (http://homer.ucsd.edu/homer/motif/HomerMotifDB/homerResults.html). Due to lack of a Nfatc2 motif in Homer motif library, the Nfatc2 motif file was downloaded from the JASPAR motif database and converted to Homer compatible format by dumpJapsar command from the monaLisa R package. Genome wide motif BED files were then overlapped with the ChIP-seq peak BED files, which were then used as the input files for computeMatrix command of the deeptools software. The computed matrix files were then used for generating the footprinting plots.

For ChIP-qPCR, pulldown DNA was incubated at 37°C with RNaseA (10 mg/ml) and protease K (0.2 mg/ml) for 2 hrs, and purified using QIAquick Spin Columns (Qiagen, #28004). qPCR was performed using iTaq Universal SYBR Green Supermix (Bio-Rad, # 1725120). The following primers sequences were used targeting the Myh8 gene:

Primer #1-F: 5′-CAACAGAAAGCTGAAGAGTG-3′

Primer #1-R: 5′-ACTGATACATCCAGTCTAGC-3′

Primer #2-F: 5′-AAGGTATGTCAAGCCTCAGC-3′

Primer #2-R: 5′-GTTTCTATGTACCCAATGTT-3′

Retroviruses generation and infection

Mouse MyoD or NFATc2 cDNA were cloned into the retroviral vector pQCXIP (Clontech, #631516). 3µg pQCXIP-empty vector or pQCXIP-MyoD or pQCXIP-NFATc2 plasmids with 1 µg pCL-Eco (Addgene, 12371) plasmids were transfected using PEI (Polysciences, #24765-1) into HEK293T (ATCC, #CRL-11268) cells at 80% confluence in a 6-well plate. 48 hours post- transfection, viral medium was collected and filtered through a 0.45 µm filter. For 3T3-L1 (ATCC, #CL-173) experiments, plated the day prior to infection at 30% confluence, and infected with viral mixture containing polybrene (Sigma-Aldrich, #H9268, 6 µg/ml) for 24 hours. Infected cells were selected with 2 µg/ml puromycin. Hypoxic cell culture was performed using a hypoxic chamber (Billups-Rothenberg) at 37°C in 5% CO_2 and 1% O_2 level.

Quantitative PCR (qRT-PCR, qPCR)

Total RNA was extracted from FACS-sorted MuSCs using the RNeasy Micro Kit (QIAGEN #74004) following the manufacturer's instructions. After RNA isolation, real-time qRT-PCR were performed with Luna Universal One-Step RT-qPCR Kit (New England Biolabs, #E3005) following the manufacturer's protocol. Transcript levels were normalized to *β2-microglobulin* (β2M) using the $2^{-\Delta\Delta CT}$ method (74). For genomic PCR reactions, genomic DNA was purified using phenol-choloroform extraction, and real time qPCR was performed with the iTAQ Universal SYBR Green Supermix (Bio-Rad, #1725120). The following primers sequences were used:

Pgc-1α-F: 5′-CCCTGCCATTGTTAAGACC-3′

Pgc-1α-R: 5′-TGCTGCTGTTCCTGTTTTC-3′

Pgc-1β-F: 5′-TCCTGTAAAAGCCCGGAGTAT-3′

Pgc-1β-R: 5′-GCTCTGGTAGGGGCAGTGA-3′

Hif-1α-F: 5′-CCTGCACTGAATCAAGAGGTTGC-3′

Hif-1α-R: 5′-CCATCAGAAGGACTTGCTGGCT-3′

Vhl-F: 5′-GTTTGTGCCATCCCTCAATGTCG-3′

Vhl-R: 5′-ACCTGACGATGTCCAGTCTCCT-3′

Mfn1α-F: 5′- ATGGCAGAAACGGTATCTCCA-3′

Mfn1α-R: 5′- CTCGGATGCTATTCGATCAAGTT-3′

Mfn2-F: 5′- TGACCTGAATTGTGACAAGCTG-3′

Mfn2-R: 5′- AGACTGACTGCCGTATCTGGT-3′

β2 Microglobulin-F: 5′-TTCTGGTGCTTGTCTCACTGA-3′

β2 Microglobulin-R: 5′- CAGTATGTTCGGCTTCCCATTC-3′

mtDNA-F: 5′- CCTATCACCCTTGCCATCAT-3′

mtDNA -R: 5′- GAGGCTGTTGCTTGTGTGAC-3′

nDNA-F: 5′- ATGGAAAGCCTGCCATCATG-3′

nDNA-R: 5′- TCCTTGTTGTTCAGCATCAC-3′

<u>Western blot analysis</u>

For 3T3-L1 (ATCC, #CL-173) experiments, trypsinized cells were spun down, washed with PBS, and resuspended in RIPA buffer (Thermo Scientific, #89900) supplemented with protease inhibitor cocktail (Roche, #11873580001), and put on ice for 30 min. Lysates were spun down at 12,000g at 4°C for 10 min. Protein concentrations were quantitated with the DC protein assay (Bio-Rad, #5000112). For MuSCs, 50,000 FACS-isolated cells were collected in PBS, spun down and resuspended in 50μL RIPA buffer,

and processed as above. For myofibers, regenerative myofibers were separated, and minced in RIPA buffer supplemented with protease inhibitor cocktail, and put on ice for 30 min. Lysates were spun down at 12,000g at 4°C for 10 min. Protein concentrations were quantitated with the DC protein assay. The following antibodies were used: Mfn1 (Proteintech, #13798-1-AP, 1:1000), Mfn2 (Proteintech, #12186-1-AP, 1:1000), Pgc-1β (Proteintech, #22378-1-AP, 1:1000), Hif-1α (Novus Biologicals, #NB100-479, 1:1000), NFATc2 (Thermo Fisher, #MA1-025, 1:1000), Histone H2B (Santa Cruz Biotechnology, #sc-515808, 1:1000). H3K9me3 (Abcam, #ab8898, 1:1000), H3K27me3 (Abcam, #ab6002, 1:1000), MyoD (Santa Cruz Biotechnology, #sc-32758, 1:1000), PDK4 (Abcam, #ab214938, 1:1000), PDH (Cell Signaling Technology, #3205, 1:1000), p-PDH (Ser293) (Cell Signaling Technology, #37115, 1:1000), KDM4A (Abcam, #ab191433, 1:1000), KDM4B (Thermo Fisher, #PA5-115460, 1:1000), KDM4C (Thermo Fisher, # PA5-23065, 1:1000), Foxm1 (Abcam, #ab207298, 1:1000), KDM6A (Abcam, #ab253183, 1:1000), KDM6B (Thermo Fisher, # PA5-72751, 1:1000), Histone3 (Abcam, # ab176840, 1:1000).

<u>Statistics</u>

No statistical tests were used to predetermine sample size, and no data were excluded. Data sets for each group of measurement were tested for normality using the Shapiro-Wilk test. If the data was not normally distributed, the data was log-transformed and retested for normality. For normally-distributed data, groups were compared using the two-tailed Student's t-test (for 2 groups), or one-way ANOVA or two-way ANOVA (> 2 groups), followed by Tukey's or Dunnett's test for multiple comparisons. For data that was not normally distributed, we used non-parametric testing (Mann-Whitney or Kolmogorov-

Smirnov tests for two groups and Kruskal-Wallis test for multiple groups), followed by Dunn's multiple comparisons adjustment. A p-value < 0.05 was considered significant. Multiple independent experiments with biological replicates were performed for all reported data, and the number of biological replicates are indicated in the figures. For representative imaging data, the experiment was conducted at least three times, with the exception of human studies where the experiment was conducted twice.

Supplementary Tables

Table S1: Supplemental data analysis for MyoD and MyoG ChIP-seq analysis in wild-type quiescent and activated muscle stem cells.

Table S2: Supplemental data analysis for H3K9me3 and H3K27me3 ChIP-seq analysis in wild-type and *mfn2*$^{-/-}$ 14 dpi myofibers.

Table S3: Expression and gene ontology statistics for differentially expressed genes in *mfn2*$^{-/-}$ vs. wild-type 2 dpi activated muscle stem cells.

Table S4: Metabolomic analysis of wild-type and *mfn2*$^{-/-}$ regenerating myofibers at 5 and 14 days post injury.

Table S5: Summary data for CNM patient and control specimens.

Figure S1

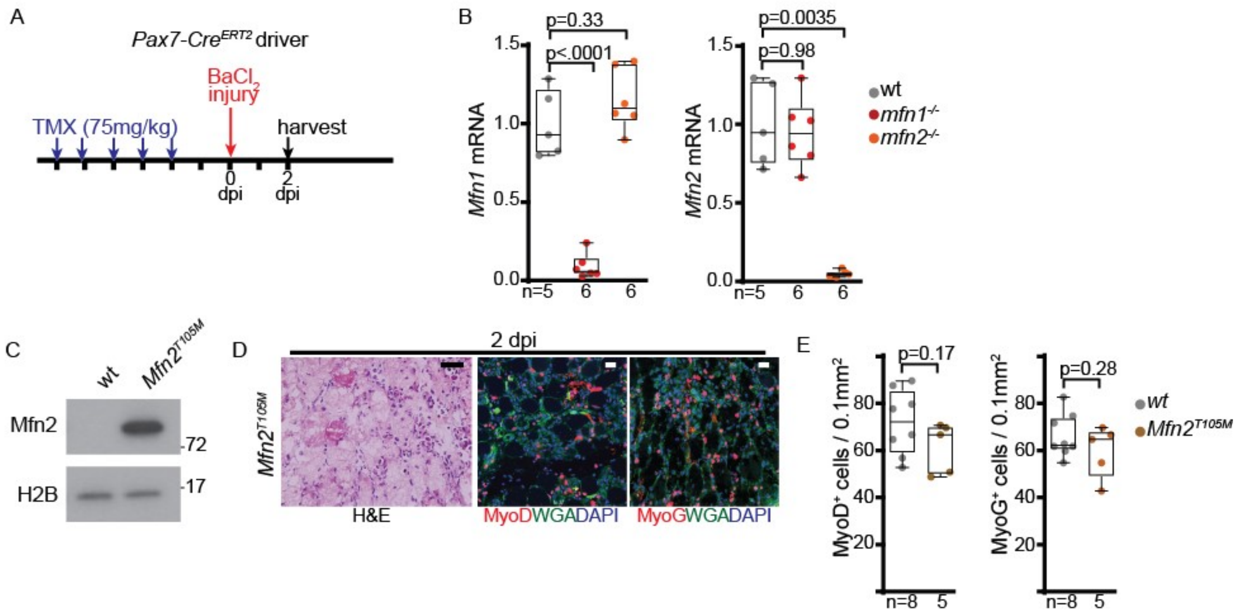

Supplementary Figure S1. Depletion of Mfn1 or Mfn2 using the *Pax7-Cre^{ERT2}* drivers. (A) Schematic to induce recombination in MuSCs using the *Pax7-Cre^{ERT2}* driver. Tamoxifen was administered for 5 consecutive days to induce recombination in QSCs, followed by $BaCl_2$ muscle injury. Activated MuSCs (ASCs) were harvested at 2 dpi. **(B)** *Mfn1* and *Mfn2* mRNA levels (relative to *β2-microglobulin*; normalized) in 2 dpi ASCs of the indicate genotype, as measured by qRT-PCR. **(C)** Mfn2 levels in 2 dpi ASCs of the indicated genotype, assessed by Western blot. Molecular weight markers (in kDa) are indicated. Histone 2B (H2B) is shown as a loading control. **(D)** Representative histology (H&E) and immunofluorescence images of muscle cross-sections from *Mfn2^{T105M}* mice of the indicated genotypes at 2 dpi. For immunofluorescence images, nuclei were visualized with DAPI (blue), myofiber borders were visualized with WGA (green), and MyoD or MyoG (red) were stained using the respective antibodies. Scale bar: 50 μm. **(E)** The number of activated stem cells (MyoD$^+$ or MyoG$^+$) from muscles of the indicated genotype at 2 dpi, normalized to cross-sectional area. Statistical significance was assessed using one way-

ANOVA (B) or two-tailed t-tests (E) with adjustments for multiple comparisons. Box plots indicate median and interquartile ranges from the indicated number of biological replicates; whiskers are plotted using the Tukey method.

Figure S2

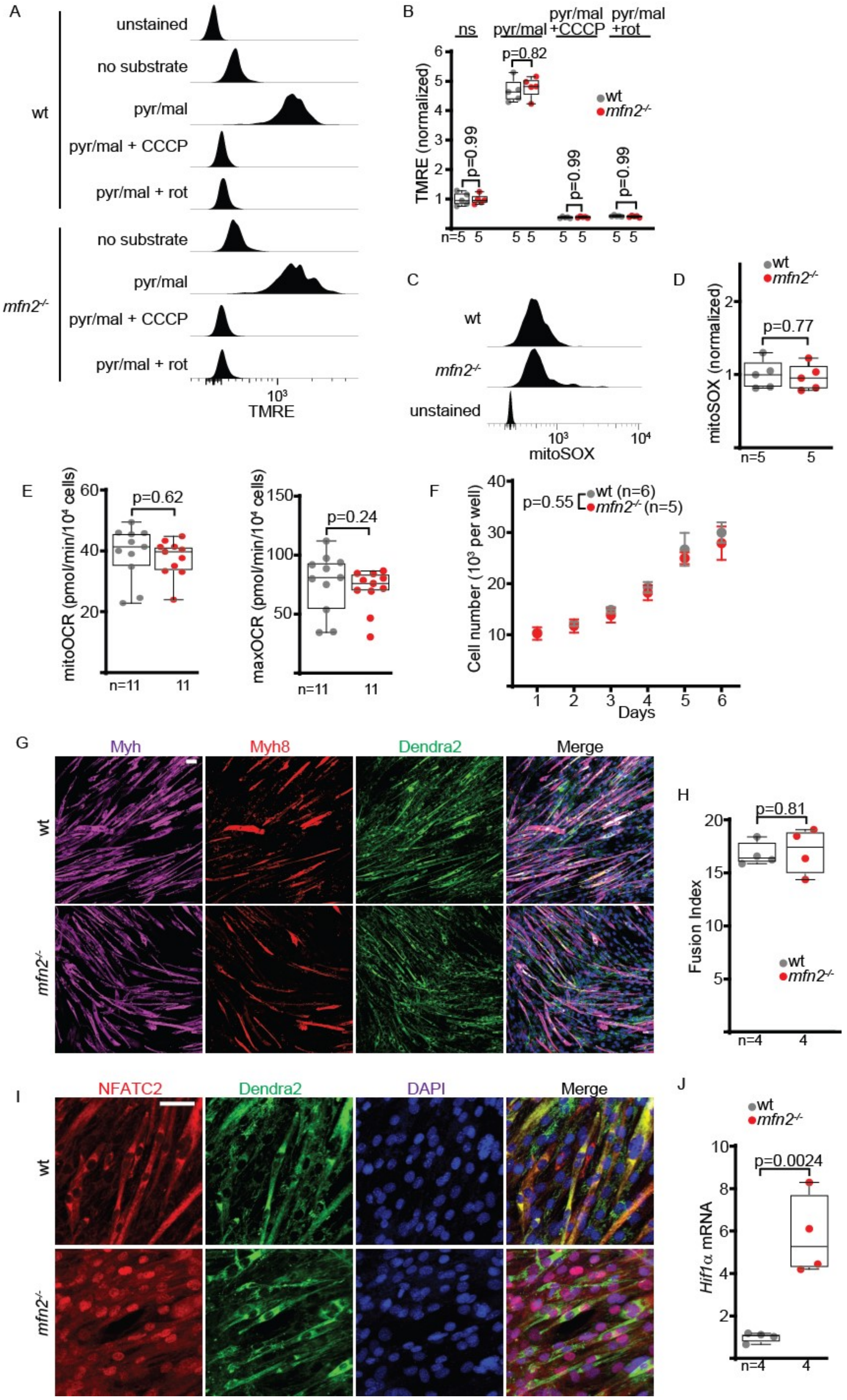

A
unstained
no substrate
wt
pyr/mal
pyr/mal + CCCP
pyr/mal + rot
no substrate
mfn2-/-
pyr/mal
pyr/mal + CCCP
pyr/mal + rot
10³
TMRE
B
ns
pyr/mal
pyr/mal +CCCP
pyr/mal +rot
p=0.82
p=0.99
p=0.99
p=0.99
TMRE (normalized)
wt
mfn2-/-
n=5 5 5 5 5 5 5 5
C
wt
mfn2-/-
unstained
10³
10⁴
mitoSOX
D
wt
mfn2-/-
mitoSOX (normalized)
p=0.77
n=5 5
E
mitoOCR (pmol/min/10⁴ cells)
p=0.62
n=11 11
maxOCR (pmol/min/10⁴ cells)
p=0.24
n=11 11
F
p=0.55
wt (n=6)
mfn2-/- (n=5)
Cell number (10³ per well)
Days
G
Myh
Myh8
Dendra2
Merge
wt
mfn2-/-
H
p=0.81
Fusion Index
wt
mfn2-/-
n=4 4
I
NFATC2
Dendra2
DAPI
Merge
wt
mfn2-/-
J
wt
mfn2-/-
p=0.0024
Hif1α mRNA
n=4 4

Supplementary Figure S2. In vitro analysis of wild-type and *mfn2*$^{-/-}$ MuSCs. (A) Representative FACS profiles of TMRE fluorescence for wild-type and *mfn2*$^{-/-}$ quiescent MuSCs in the indicated buffers. pyr/mal, pyruvate/malate. rot, rotenone. CCCP, carbonyl cyanide m-chlorophenyl hydrazone. **(B)** Mean TMRE fluorescence (normalized) of wild-type and *mfn2*$^{-/-}$ MuSCs in the indicated buffers. **(C)** Representative FACS profiles of mitoSOX fluorescence for quiescent MuSCs of the indicated genotype. **(D)** Mean mitoSOX fluorescence (normalized) for MuSCs of the indicated genotype. **(E)** Basal mitochondrial oxygen consumption rates (OCR) and maximal (CCCP-stimulated) OCR for MuSCs of the indicated genotype. **(F)** In vitro proliferation of MuSCs of the indicated genotype. **(G)** Representative immunofluorescent images of differentiated myotubes (in vitro) from the indicated genotype. Myosin heavy chain (Myh), neonatal myosin heavy chain (Myh8), mitochondria (Dendra2) and nuclei (DAPI) are visualized in the indicated colors. Scale bar, 50 μm. **(H)** Fusion indices of differentiated MuSCs (in vitro) from the indicated genotype. **(I)** Representative immunofluorescent images of differentiated myotubes of the indicated genotype. NFATC2, mitochondria (Dendra2), nuclei (DAPI) are visualized in the indicated colors. Scale bar, 20 μm. **(J)** *Hif1*α mRNA transcript levels (relative to β2microglobulin; normalized) from in vitro myotubes of the indicated genotype measured by qRT-PCR. Statistical significance was assessed using two way-ANOVA (B,F), two-tailed t-tests (D,E,H,J), two-tailed Mann-Whitney tests (E) with adjustments for multiple comparisons. Box plots indicate median and interquartile ranges from the indicated number of biological replicates; whiskers are plotted using the Tukey method.

Figure S3

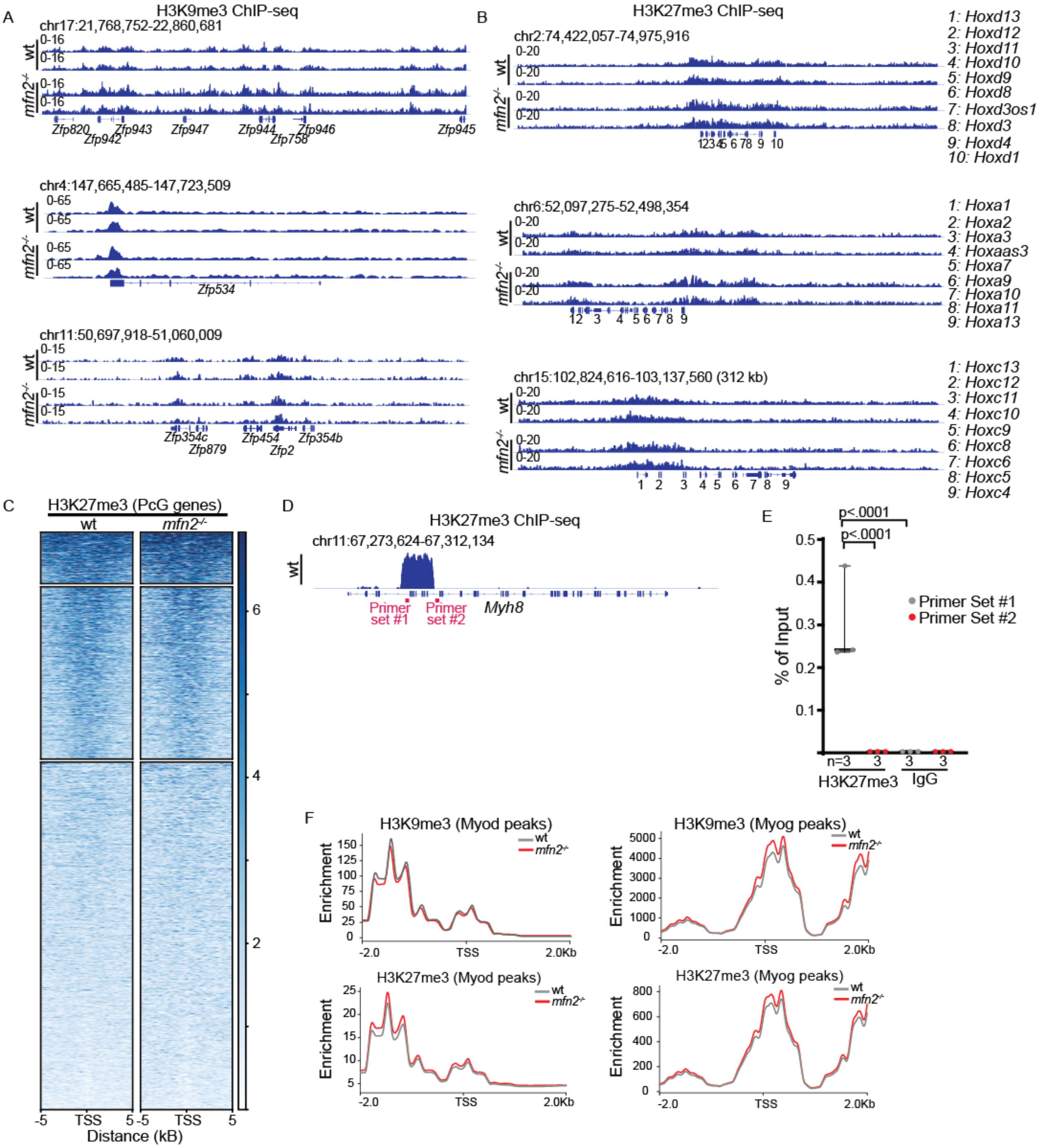

Supplementary Figure S3. Analysis of H3K9me3 and H3K27me3 ChIP-seq data in 14 dpi myofibers. (A) Representative snapshots of H3K9me3 ChIP-seq analysis at the indicated zinc finger transcription factor loci, in 14 dpi myofibers of the indicated genotype.

(B) Representative snapshots of H3K27me3 ChIP-seq analysis at the indicated Hox transcription factor loci, in 14 dpi myofibers of the indicated genotype. **(C)** Heatmaps representing normalized H3K27me3 ChIP-seq intensities of PcG target genes in 14 dpi myofibers of the indicated genotype, after k-means clustering. Peaks were ranked according to their ChIP-seq intensity in wild-type samples. n=3 mice per group. **(D)** Representative snapshots of H3K9me3 ChIP-seq analysis at the *Myh8* gene, in 14 dpi wild-type myofibers. Positions of primer set #1 and #2 (used in panel e) are indicated. **(E)** Enrichment (% of input) from ChIP-qPCR experiments using H3K27me3 or control (IgG) antibodies. Two different primer sets targeting the *Myh8* gene were used. Primer set #1 was located within the H3K27me3 peak; primer set #2 is located outside the H3K27me3 peak. **(F)** Footprinting plots of H3K9me3 and H3K27me3 deposition in wild-type and *mfn2*$^{-/-}$ 14 dpi myofibers at MyoD and MyoG peaks identified in ASCs (Figure 1C; Table S1). Statistical significance was assessed using two tailed t-tests (E) with adjustments for multiple comparisons. For each ChIP-seq dataset, three biological replicates were analyzed. Box plots indicate median and interquartile ranges from the indicated number of biological replicates; whiskers are plotted using the Tukey method.

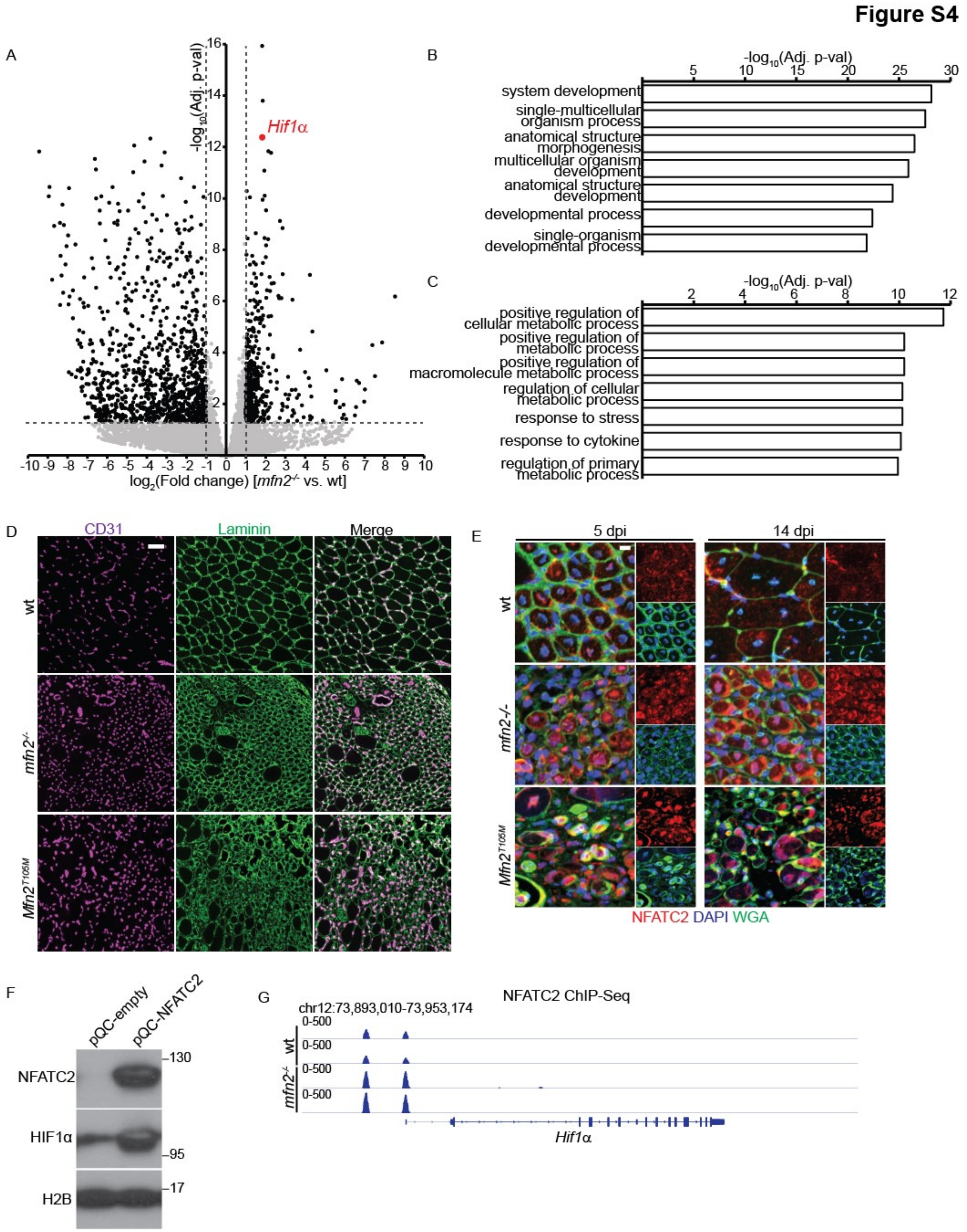

Supplementary Figure S4. Elevated Hif1α and NFATc2 signaling in *mfn2*$^{-/-}$ regenerating myofibers. (A) Volcano plot of gene expression changes in 2 dpi *mfn2*$^{-/-}$

vs. wild-type activated MuSCs (ASCs), based on RNAseq analysis. Log_2(Fold change) is potted against the $-log_{10}$(adjusted p-value) for each gene. The position of *Hif1α* is highlighted in red. **(B)** The top enriched biological pathways from gene ontology analysis of down-regulated genes (log_2(Fold change) < -1; Adjusted p-value < 0.05) in *mfn2*$^{-/-}$ ASCs. **(C)** The top enriched biological pathways from gene ontology analysis of up-regulated genes (log_2(Fold change) > 1; Adjusted p-value < 0.05) in *mfn2*$^{-/-}$ ASCs. **(D)** Representative immunofluorescence images from muscle cross sections of the indicated genotype at 14 dpi. Sections were stained for endothelial cells (CD31, purple) and myofiber boundaries (laminin, green). Scale bar, 50 μm. **(E)** Representative immunofluorescence images from muscle cross-sections of the indicated genotype at 5 and 14 dpi. Sections were stained for NFATC2 (red), DAPI (blue) or myofiber boundaries (WGA; green). Scale bar, 10μm. **(F)** Mouse 3T3-L1 fibroblasts were infected with retrovirus made with empty vector (pQC-empty) or *NFATC2* expressing vector (pQC-NFATC2), followed by hypoxia treatment (1% O2) for 24 hours. The indicated targets were assessed by western blot. Histone 2B (H2B) is shown as a loading control. Molecular weight markers (in kDa) are indicated. **(G)** Representative snapshots of NFATc2 binding at the *Hif1α* promoter, in 14 dpi wild-type myofibers of the indicated genotype. For each ChIP-seq dataset, 2 biological replicates were analyzed. For each RNAseq dataset, 4 biological replicates were analyzed.

1557

Figure S5

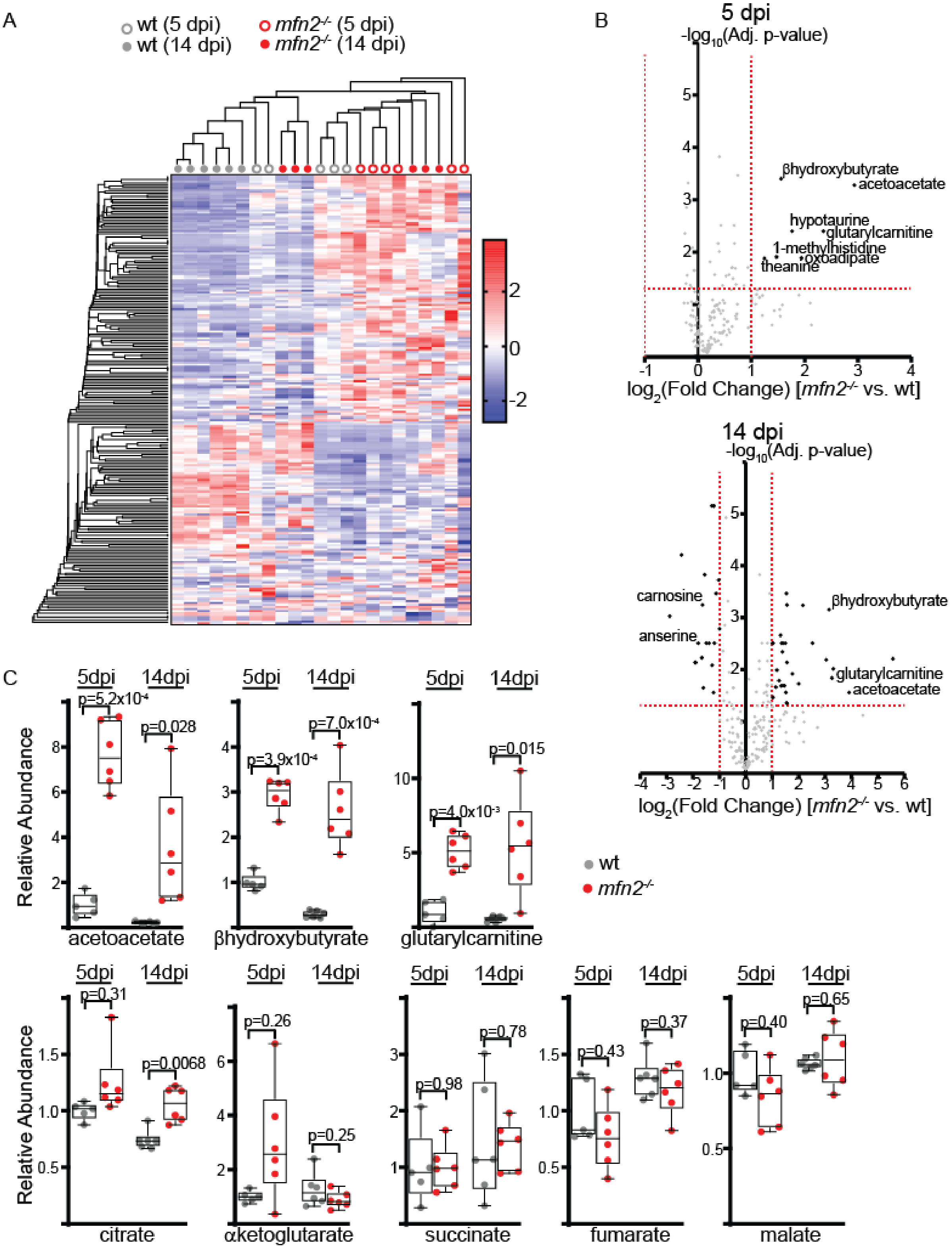

Supplementary Figure S5. Metabolic analysis of wild-type and *mfn2*$^{-/-}$ regenerating myofibers. (A) Unsupervised hierarchical clustering of metabolite profiles from wild-type and *mfn2*$^{-/-}$ myofibers at 5 and 14 dpi. Individual pixels are colored by z-score according to the indicated colorbar. n=5-6 mice per group. **(B)** Volcano plots comparing the relative fold change in metabolite abundance between wt and *mfn2*$^{-/-}$ myofibers at 5 dpi (top) and 14 dpi (bottom). Metabolites shown in black were significantly changed (adjusted p-value < 0.05; $|\log_2(\text{Fold change})| > 1$). **(C)** Relative metabolite abundances of the selected TCA cycle and ketogenic metabolites in myofibers of the indicated genotype and 5 and 14 dpi. n=5-6 mice per group. Statistical significance was assessed using two tailed t-tests (B,C) with adjustments for multiple comparisons. Box plots indicate median and interquartile ranges from the indicated number of biological replicates; whiskers are plotted using the Tukey method.

Figure S6

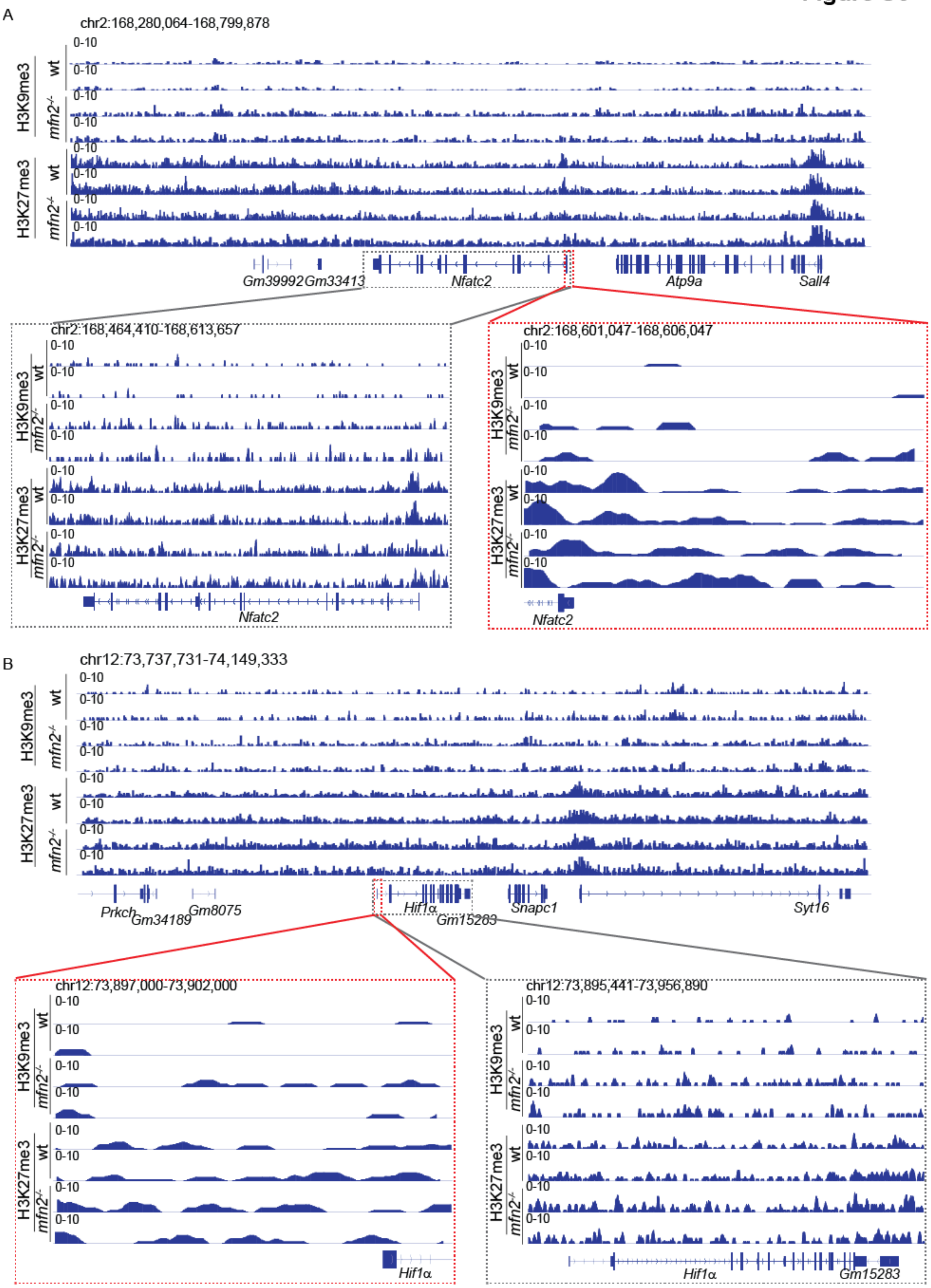

1582

Supplementary Figure S6. H3K9me3 and H3K27me3 profiles in wild-type and *mfn2*$^{-/-}$ myofibers. (A) Representative snapshots of H3K9me3 and H3K27me3 profiles at the *Nfatc2* locus. Enlarged views are shown for the entire gene body (gray) and the promoter region (red). **(B)** Representative snapshots of H3K9me3 and H3K27me3 profiles at the *Hif1*α locus. Enlarged views are shown for the entire gene body (gray) and the promoter region (red). For each ChIP-seq dataset, 3 biological replicates were analyzed.

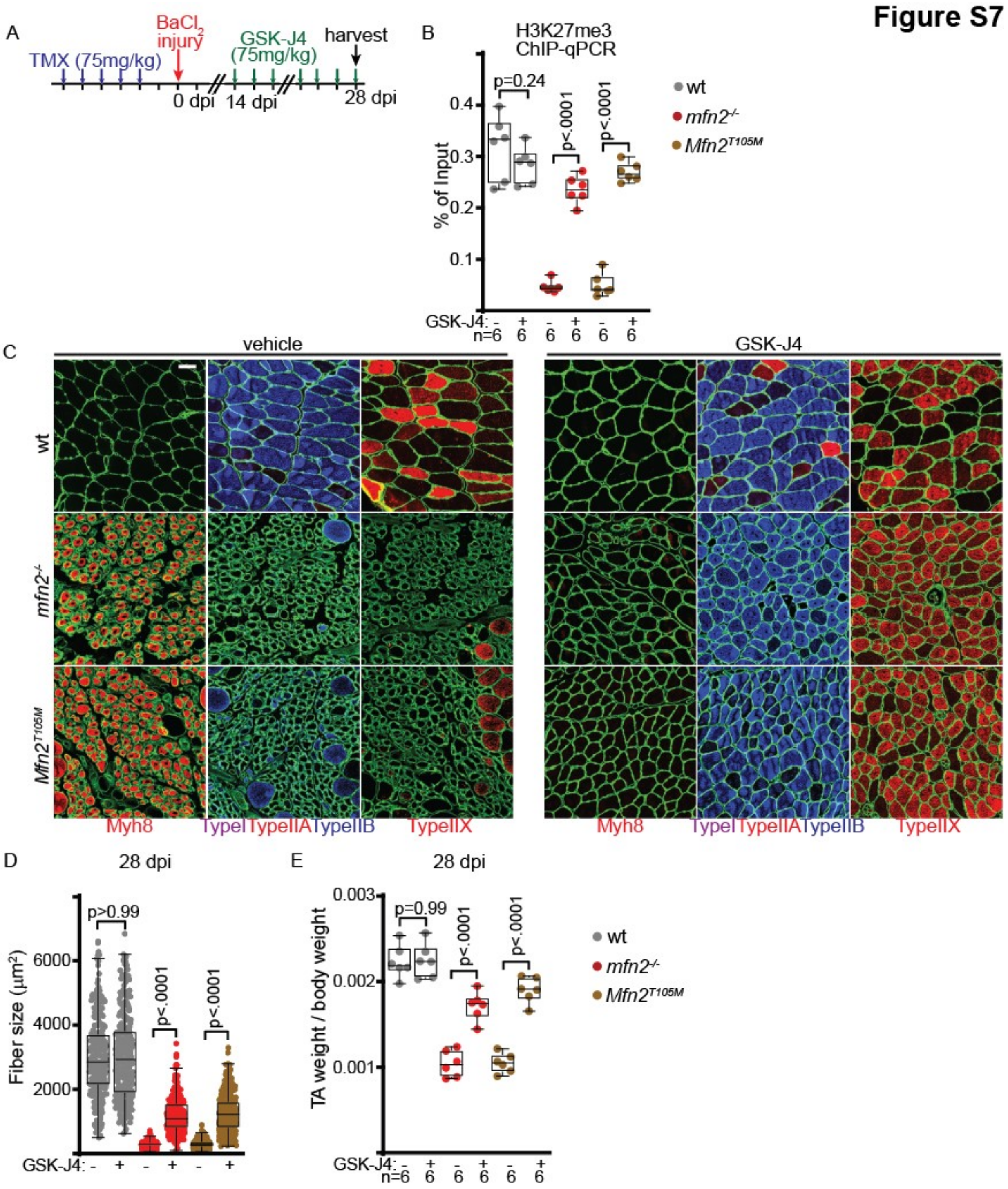

Supplementary Figure S7. Inhibition of H3K27me3 demethylases allows Mfn2-mutant myofibers to adopt adult fates. (A) Schematic of GSK-J4 experiment. Tamoxifen (TMX) was administered for 5 consecutive days to induce recombination,

followed by $BaCl_2$ administration to injure muscles. At 14 dpi, mice were subsequently treated with GSK-J4 (or vehicle) every day. Tissue was harvested at 28 dpi for analysis. **(B)** Quantitation of enrichment (% of input) from H3K27me3 ChIP-qPCR experiments targeting the *Myh8* gene. Experiments were performed in 28 dpi myofibers from animals of the indicated genotype and treatment condition. **(C)** Representative immunofluorescence images of muscle cross-sections from mice of the indicated genotype and treatment condition at 28 dpi. Sections were stained with antibodies targeting fiber-type specific myosin heavy chains, including Myh7 (type I; purple), Myh2 (type IIa; red), Myh4 (type IIb; blue), Myh1 (type IIx; red), and Myh8 (neonatal; red). Myofiber borders were visualized with Laminin staining (green). Scale bar, 50 μm. **(D)** Cross-sectional area of regenerating fibers from muscles of the indicated genotype and treatment conditions at 28 dpi. 300 myofibers were analyzed from n=6 mice per group. **(E)** Tibialis anterior (TA) muscle weight (normalized to body weight) from mice of the indicated genotype and treatment condition at 28 dpi. Statistical significance was assessed using two-way ANOVA (B,E), or Kruskal-Wallis (D) tests with adjustments for multiple comparisons. Box plots indicate median and interquartile ranges from the indicated number of biological replicates; whiskers are plotted using the Tukey method.

Figure S8

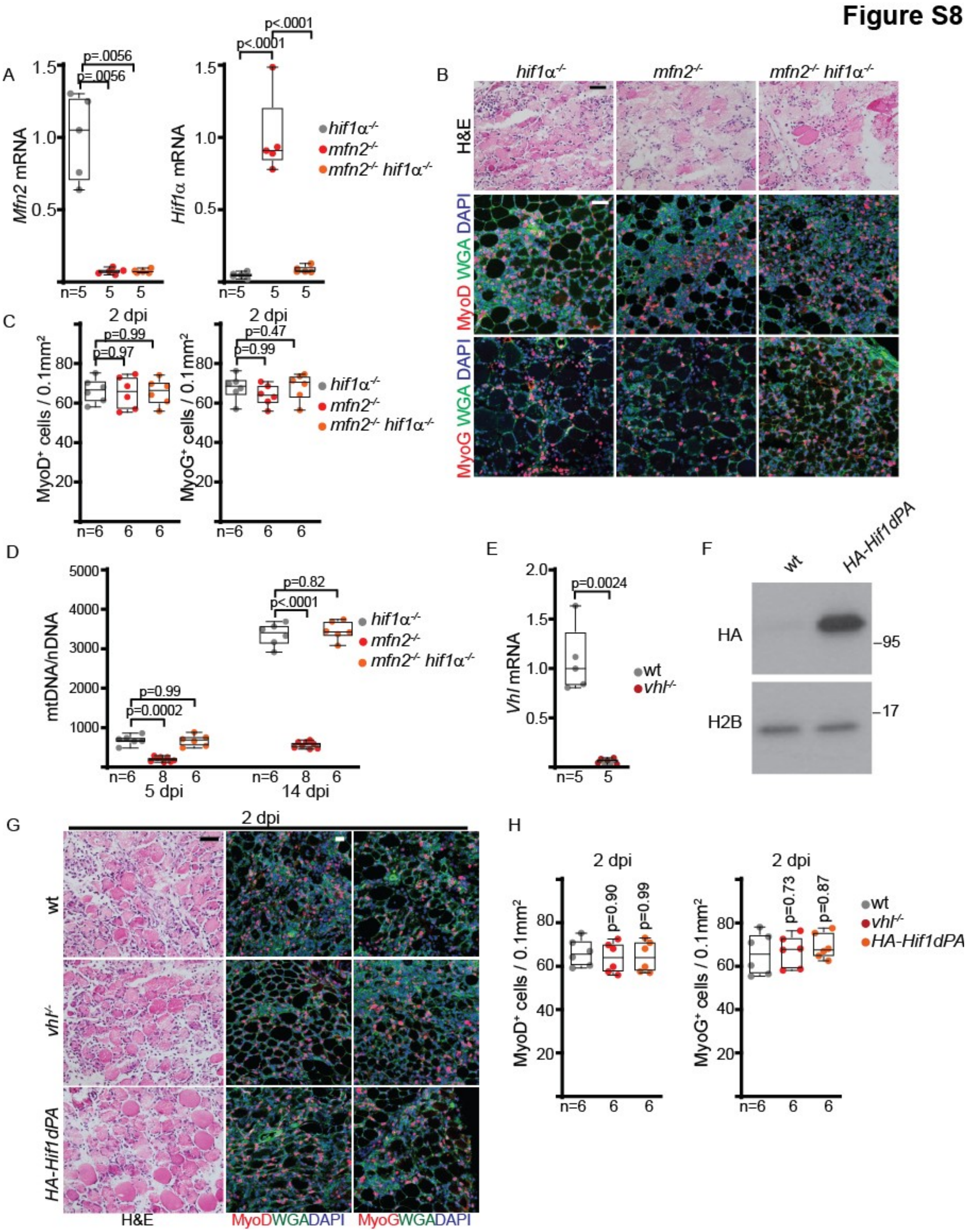

Supplementary Figure S8. Hif1α is not required for activation of MuSCs. (A) *Mfn2* and *Hif1α* mRNA levels (relative to *β2-microglobulin*; normalized) in 2 dpi ASCs of the

indicated genotype, as measured by qRT-PCR. **(B)** Representative histology (H&E) and immunofluorescence images of muscle cross-sections from mice of the indicated genotypes at 2 dpi. For immunofluorescence images, nuclei were visualized with DAPI (blue), myofiber borders were visualized with laminin staining (green), and MyoD or MyoG (red) were stained using their respective antibodies. Scale bar, 50 μm. **(C)** The number of activated stem cells (MyoD$^+$ or MyoG$^+$) from muscles of the indicated genotype at 2 dpi, normalized to cross-sectional area. **(D)** Mitochondrial genome (mtDNA) content, normalized to nuclear genome content (nDNA) in 5 and 14 dpi myofibers of the indicated genotype, assessed by qPCR. **(E)** *Vhl* mRNA levels (relative to β2-microglobulin; normalized) in 2 dpi ASCs of the indicated genotype, assessed by qRT-PCR. **(F)** HA-Hif1dPA protein levels in 2 dpi ASCs of the indicated genotype, assessed by Western blot. Molecular weight markers (in kDa) are indicated. Histone 2B (H2B) is shown as a loading control. **(G)** Representative histology (H&E) and immunofluorescence images of muscle cross-sections from mice of the indicated genotypes at 2 dpi. For immunofluorescence images, nuclei were visualized with DAPI (blue), myofiber borders were visualized with laminin staining (green), and MyoD or MyoG (red) were stained using their respective antibodies. Scale bar, 50 μm. **(H)** The number of activated stem cells (MyoD$^+$ or MyoG$^+$) from muscles of the indicated genotype at 2 dpi, normalized to cross-sectional area. p-values indicate comparisons with the wild-type group. Statistical significance was assessed using one-way ANOVA (A,C,H), two-tailed t-test (E) or two-way ANOVA (D) with adjustments for multiple comparisons. Box plots indicate median and interquartile ranges from the indicated number of biological replicates; whiskers are plotted using the Tukey method.

Figure S9

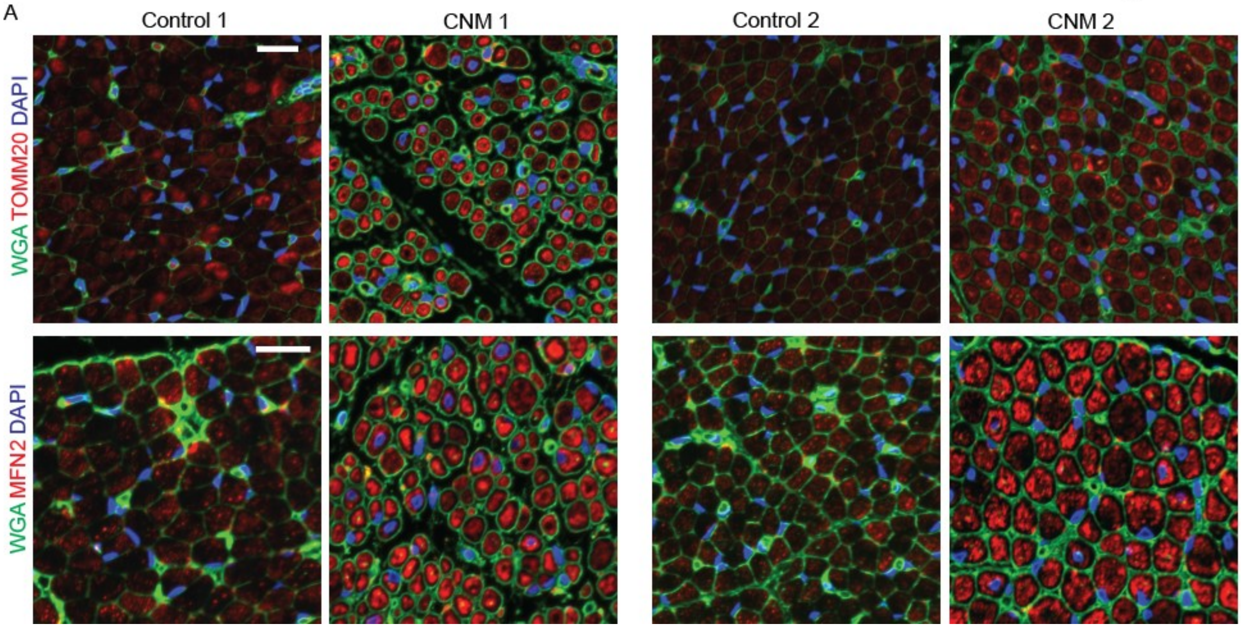

Supplementary Figure S9: Peri-nuclear mitochondrial localization in CNM muscle specimens. (A) Representative immunofluorescence images for TOMM20 (mitochondria), or MFN2 from a patient with infantile CNM due to a *Dnm2* mutation (CNM1) and an age/sex-matched control (Control 1), and a patient with infantile CNM due to a *Mtm1* mutation (CNM2) and an age/sex-matched control (Control 2). Myofiber boundaries are visualized with WGA staining, and nuclei are visualized with DAPI staining. Scale bar, 20 μm.

Figure S10

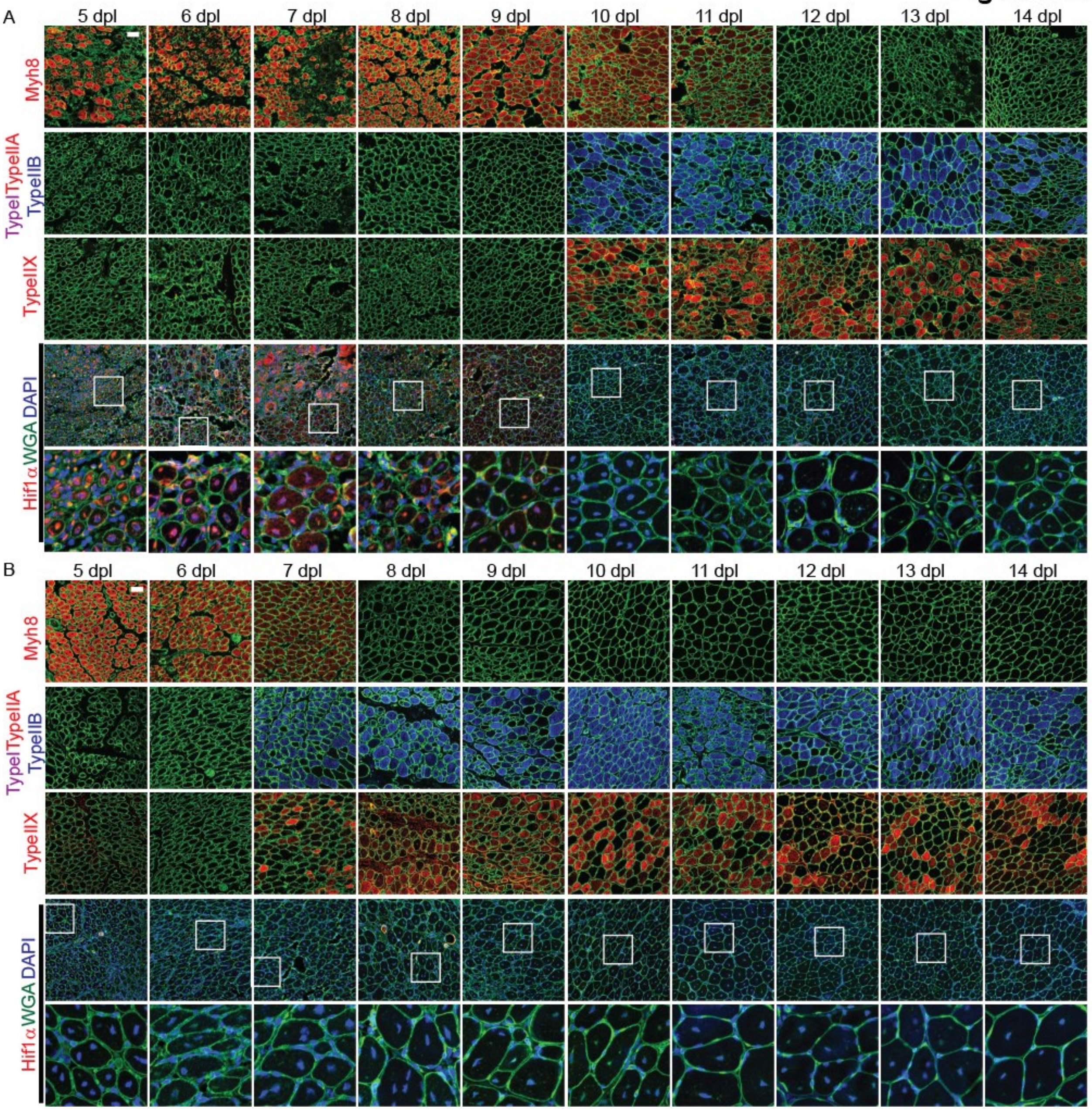

Supplementary Figure S10. Regenerating myofibers pause at the neonatal-adult transition in response to ischemic injury. (A) Representative immunofluorescence images of muscle cross-sections from wild-type mice at the indicated time point. Muscle cross-sections were stained with antibodies targeting fiber-type specific myosin heavy chains, including Myh7 (type I; purple), Myh2 (type IIa; red), Myh4 (type IIb; blue), Myh1

(type IIx; red), and Myh8 (neonatal; red), or Hif1α (red). Nuclei were visualized with DAPI, and myofiber boundaries were visualized with Laminin staining (green) or WGA (green). Scale bar: 50 μm. dpl, days post ligation. **(B)** Same as (a), except with *hif1α*$^{-/-}$ animals.

Figure S11

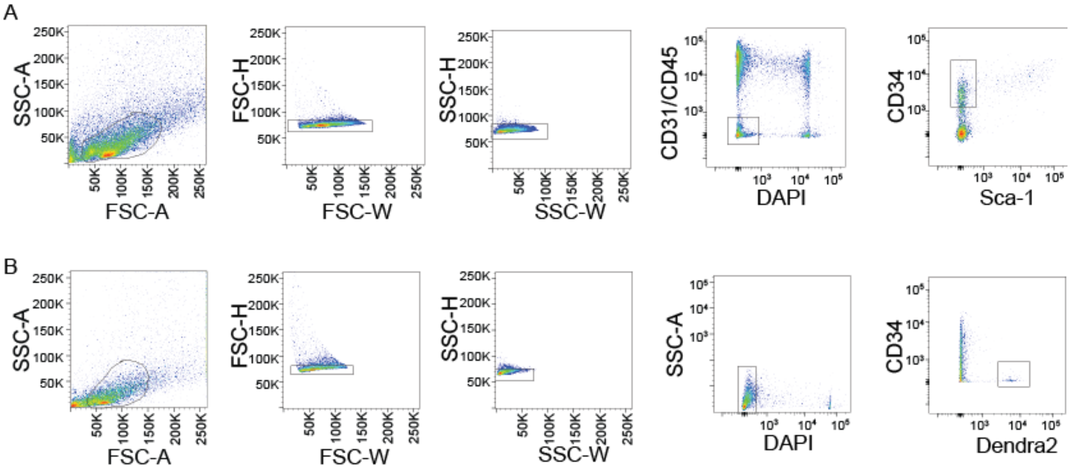

Supplementary Figure S11: Representative gating strategy for MuSC isolation. (A) Representative gating strategy for quiescent MuSCs. **(B)** Representative gating strategy for activated MuSCs.

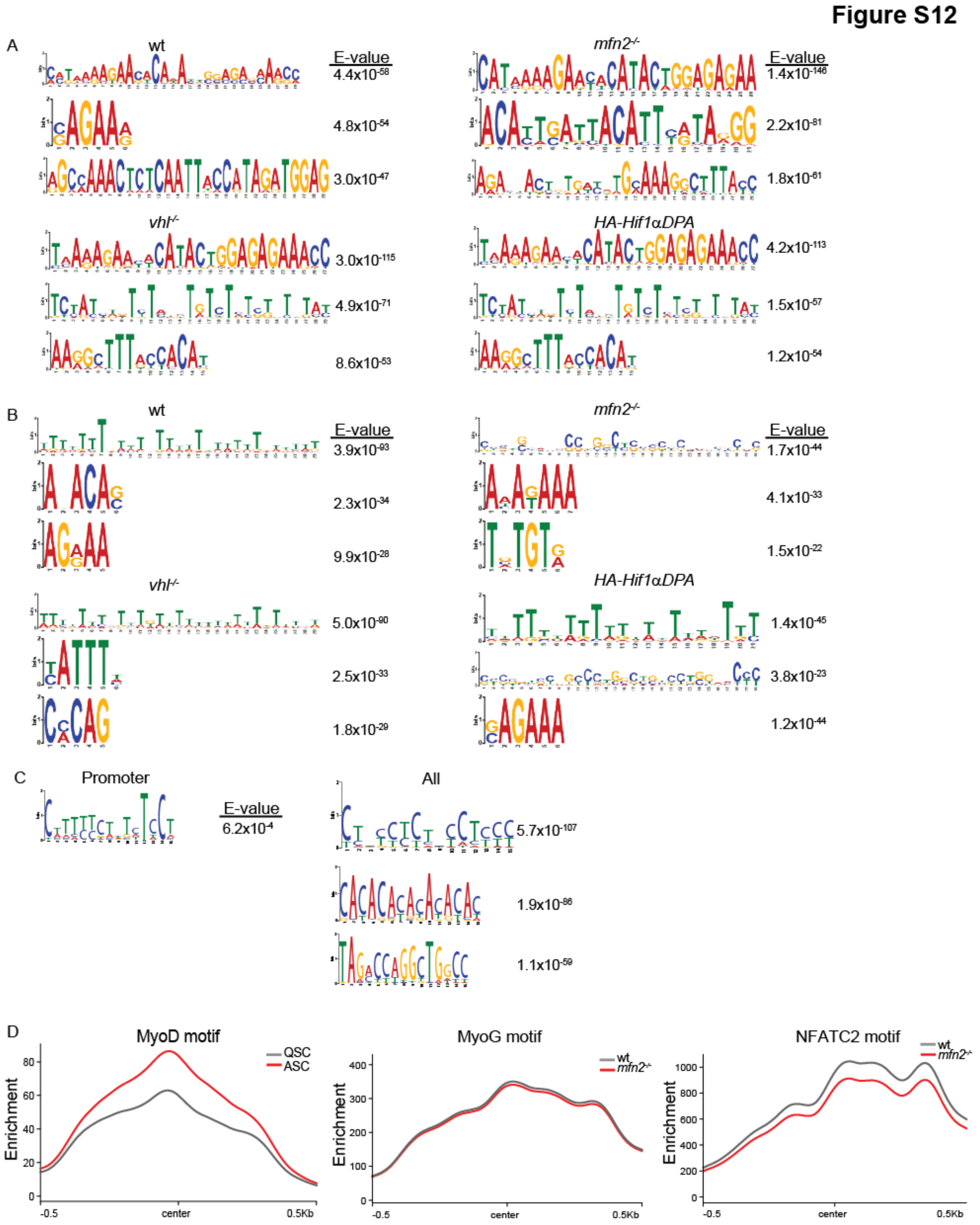

Supplementary Figure S12. Motif analysis for ChIP-seq datasets. (A) Top three enriched motifs for H3K9me3 peaks in 14 dpi myofibers of the indicated genotypes. E-

values are indicated for each motif. **(B)** Same as (A), except for H3K27me3 peaks. **(C)** Top enriched motifs for H3K27me3 differentially expressed peaks between wt and *mfn2*$^{-/-}$ myofibers in either promoter regions (“Promoter”) or genome-wide (“All”). **(D)** Signal enrichment curves for transcription factor binding to their respective consensus binding sequences in the indicated data set. For each ChIP-seq dataset, 3 biological replicates were analyzed, except for NFATC2 binding, where 2 biological replicates were analyzed.
